# A Machine-Learning-Based Risk-Prediction Tool for HIV and Sexually Transmitted Infections Acquisition over the Next 12 Months

**DOI:** 10.3390/jcm11071818

**Published:** 2022-03-25

**Authors:** Xianglong Xu, Zongyuan Ge, Eric P. F. Chow, Zhen Yu, David Lee, Jinrong Wu, Jason J. Ong, Christopher K. Fairley, Lei Zhang

**Affiliations:** 1 Melbourne Sexual Health Centre, Alfred Health, Melbourne, VIC 3053, Australia; xianglong.xu@monash.edu (X.X.); echow@mshc.org.au (E.P.F.C.); dlee@mshc.org.au (D.L.); doctorjasonong@gmail.com (J.J.O.); christopher.fairley@monash.edu (C.K.F.); 2 Central Clinical School, Faculty of Medicine, Nursing and Health Sciences, Monash University, Melbourne, VIC 3800, Australia; zhen.yu@monash.edu; 3 China Australia Joint Research Center for Infectious Diseases, School of Public Health, Xi’an Jiaotong University Health Science Centre, Xi’an 710061, China; 4 Monash e-Research Centre, Faculty of Engineering, Airdoc Research, Nvidia AI Technology Research Centre, Monash University, Melbourne, VIC 3800, Australia; zongyuan.ge@monash.edu; 5 Centre for Epidemiology and Biostatistics, Melbourne School of Population and Global Health, The University of Melbourne, Melbourne, VIC 3053, Australia; 6 Research Centre for Data Analytics and Cognition, La Trobe University, Bundoora, VIC 3086, Australia; melody.wu@latrobe.edu.au; 7 Department of Epidemiology and Biostatistics, College of Public Health, Zhengzhou University, Zhengzhou 450001, China

**Keywords:** HIV, sexually transmitted infections, machine learning, risk prediction, behavioural intervention

## Abstract

Background: More than one million people acquire sexually transmitted infections (STIs) every day globally. It is possible that predicting an individual’s future risk of HIV/STIs could contribute to behaviour change or improve testing. We developed a series of machine learning models and a subsequent risk-prediction tool for predicting the risk of HIV/STIs over the next 12 months. Methods: Our data included individuals who were re-tested at the clinic for HIV (65,043 consultations), syphilis (56,889 consultations), gonorrhoea (60,598 consultations), and chlamydia (63,529 consultations) after initial consultations at the largest public sexual health centre in Melbourne from 2 March 2015 to 31 December 2019. We used the receiver operating characteristic (AUC) curve to evaluate the model’s performance. The HIV/STI risk-prediction tool was delivered via a web application. Results: Our risk-prediction tool had an acceptable performance on the testing datasets for predicting HIV (AUC = 0.72), syphilis (AUC = 0.75), gonorrhoea (AUC = 0.73), and chlamydia (AUC = 0.67) acquisition. Conclusions: Using machine learning techniques, our risk-prediction tool has acceptable reliability in predicting HIV/STI acquisition over the next 12 months. This tool may be used on clinic websites or digital health platforms to form part of an intervention tool to increase testing or reduce future HIV/STI risk.

## 1. Introduction

HIV and sexually transmitted infections (STIs) are global public health concerns [1,2]. The World Health Organization (WHO) estimates that more than one million people acquire an STI every day. Given the rising rates of STIs, the WHO proposed the *Global health sector strategy on Sexually Transmitted Infections, 2016–2021*, to end STI epidemics as public health concerns by 2030, which included a 90% reduction in gonorrhoea incidence globally (2018 global baseline) and less than 50 cases of congenital syphilis per 100,000 live births in 80% of countries [3]. In 2018, the 2030 agenda for sustainable development called for an end to the AIDS epidemic by 2030 [4]. One key strategy to reduce the incidence of HIV/STIs is to increase testing [5,6,7]. Barriers to testing include poor perception of HIV/STI risk, limited availability of testing, and cost [8]. Additionally, a delayed HIV diagnosis was also a common problem and caused numerous adverse health consequences [9,10]. Web-based apps for screening could effectively increase the uptake of health screening [11] and have usability and acceptability among users [12]. Predicting an individual’s future risk of HIV/STIs could contribute to behaviour change or improve testing. To the best of our knowledge, no web-based prediction tool has yet been developed to predict an individual’s risk of acquiring HIV/an STI over the next 12 months.

Machine learning algorithms have advantages for developing predictive models, such as not requiring statistical inferences or assumptions, being data driven, automatically learning from data that identifies complex nonlinear patterns, and exploiting complex interactions between risk factors [13]. Machine learning models have been used to predict the future risk of other conditions such as suicide [14,15], type 2 diabetes [16], Alzheimer’s disease [17], and myocardial infarction [18]. Two studies using electronic health-record data from the USA reported that machine learning could accurately predict future HIV infection. A study in Massachusetts, USA, reported that models using routinely collected data from electronic health records (EHR) and machine learning could accurately predict the one-year risk of acquiring HIV [19]. Another study from Kaiser Permanente Northern California, USA, reported that by using machine learning, EHR-based HIV-risk models could accurately predict an incident HIV diagnosis within three years based on 81 predictors [20]. However, none of these models have been translated into a risk-prediction tool for predicting HIV over the next 12 months. Although a few studies have been conducted on future HIV prediction, no research has been published using machine learning methods to predict syphilis, gonorrhoea, and chlamydia acquisition over the next 12 months among males and females.

The purpose of this study was to use machine learning models, including bagging, boosting, and stacking algorithms [21], and routinely collected data in the clinical settings to predict HIV and three common STIs (syphilis, gonorrhoea, and chlamydia) acquisition over the next 12 months among males and females.

## 2. Materials and Methods

### 2.1. Study Data for 12-Month HIV/STI Risk-Prediction Tool Development

We used EHR data at the Melbourne Sexual Health Centre (MSHC) to develop and validate the machine learning model. The MSHC is the largest public sexual health centre in Melbourne, Australia. In the MSHC, individuals’ demographic information, sexual practices, overseas sexual contact, and history of engaging in sex work are recorded at each visit using a computer-assisted self interview [22]. We used data from 2 March 2015 to 31 December 2019. We did not include data from 2020 because the COVID-19 epidemic could have changed the re-testing patterns and sexual practices of those attending the MSHC [23,24]. Transgender people and individuals aged below 18 years were excluded. The study was approved by the Alfred Hospital Ethics Committee, Australia (Project Number: 124/18). All methods were carried out following the relevant guidelines and regulations of the Alfred Hospital Ethics Committee.

A new diagnosis of HIV was based on serology and required a previous negative test. A diagnosis of syphilis was based on a clinician classifying the infection as early syphilis (primary, secondary, and early latent (<2 years)) using serology or a polymerase chain reaction (PCR). A diagnosis of gonorrhoea was based on a culture or a nucleic acid amplification test (NAAT) at one or more anatomical sites. A diagnosis of chlamydia was based on an NAAT at one or more anatomical sites. Our analysis included 65,043 consultations that had tested for HIV, 56,889 consultations for syphilis, 60,598 consultations for gonorrhoea, and 63,529 consultations for chlamydia. For the syphilis, gonorrhoea, and chlamydia analysis, the detailed inclusions and exclusions are in Tables S1a,b. Details of the data-cleaning procedure are provided in the Supplementary File.

### 2.2. Predictors for 12-Month HIV/STI Risk Prediction

We extracted routinely collected data from the EHR, including self-reported questions at the first visit for each visit interval (described below). The feature selection was informed by the literature review, expert opinion, and previous work [25]. This baseline predictor data for modelling included gender, age (≥18 years old), country of birth, sexual practices (e.g., had sex with a sex partner in the last 12 months, number of sex partners in the last 12 months), condom use with sex partners in the last 12 months, pre-exposure prophylaxis (PrEP) use, presenting with STI symptoms, living with HIV (for STI prediction), and reported sexual contact with partners with an STI (gonorrhoea, chlamydia, or syphilis) (summarised in Table 1 and Table S2).

### 2.3. Model Development and Training for Building a 12-Month HIV/STI Risk-Prediction Tool

We established a series of linear and nonlinear machine learning models that involved Regression Algorithms, including Multivariate Logistic Regression (MLR) and Elastic-Net Regression (ENR); Support Vector Machine Algorithms, including the Linear Support Vector Machines (without kernel extensions) (SVM (Linear)), SVM with a Polynomial Basis Kernel (Kernel SVM (Polynomial)), and SVM with a Radial Basis Function Kernel (Kernel SVM (RBF)); Bagging Ensemble Algorithms, including the Bagged Flexible Discriminant Analysis (Bagged FDA), Bagged Flexible Discriminant Analysis using Generalised Cross Validation (Bagged FDA using gCV Pruning), Bagged Multivariate Adaptive Regression Splines using Generalised Cross Validation (Bagged MARS using gCV Pruning), Random Forest (RF), and Conditional Inference Random Forest (CIRF); Boosting Ensemble Algorithms, including the Boosted Generalised Linear Model (Boosted GLM), Gradient Boosting Machines (GBM), and eXtreme Gradient Boosting (XGBoost). Based on our unpublished work, we also built a stacking model with 3 base models: ENR+GBM+RF. We also developed Naïve Bayes (NB), K-Nearest Neighbour (KNN), and multi-layer perceptron (MLP). MLR, ENR, GBM, RF, NB, MLP, and the stacking ensemble learning model (ENR+GBM+RF) were built using the h2o package. The bagged FDA, bagged FDA using gCV Pruning, and bagged MARS using gCV Pruning was built using the *earth* package. CIRF was built using the *party* package. The Boosted GLM was built using the *mboost* package. XGBoost was built using the *xgboost* package. KNN was built using the *kknn* package. The SVM(Linear) were built using the *e1071* package. The Kernel SVM (Polynomial) and Kernel SVM (RBF) were built using the *kernlab* package.

We used random-forest-based imputation to handle the missing data. The random-forest-based imputation was built using the *mice* package in R. Our machine learning models used a one-hot encoding scheme on the category variables. We used the nested cross validation (five outer folds, ten inner folds) method for the STI models to better estimate the generalisation error and solve the overfitting and selection bias caused by using a single dataset for the model selection and model training [26]. The external cross validation loop was repeated five times to solve the variance caused by the choice of the dataset to split. The prevalence of each of the four infections was below 10%, which means the data were imbalanced. Imbalanced data may cause either over-fitted or under-performed prediction results [27]. We used random under sampling in the training dataset to address the data imbalance to solve the class imbalance problem. Furthermore, an inner 10-fold CV loop was created for each model to select the tuning hyper-parameters for the maximised area under the ROC curve (AUC) on the training fold [28]. For the HIV models, we used an 80:20 random under-sampling split based on the outcome (HIV infection status) to create a training dataset and testing dataset for the analysis due to only 0.1% of consultations having a positive HIV result. All of the HIV models were trained using the training dataset with a ten-fold cross validation method and assessed the model performance on the testing dataset. Considering our datasets had data imbalances, the performance of the machine learning models was evaluated with the area under the receiver operating characteristic curve (AUC) and F1 score. Besides, we used the variable importance analysis of HIV, syphilis, gonorrhoea, and chlamydia to estimate the contribution of each of the predictors for the four infections.

The machine learning models and statistical analyses were conducted with R 3.6.1 and R Studio 1.2.5019. We used frequencies, percentages, the median, and the interquartile range (IQR) to present the descriptive analysis. We used Poisson regression to calculate incidence rates. We used MATLAB R 2019a (The Mathworks, Natick, MA, USA) to plot figures.

### 2.4. Twelve-Month HIV/STI Risk Estimate

We used the machine learning model output probability to calculate the HIV/STI risk over the next 12 months. Our machine learning models predicted the probability of HIV/an STI with a normalised distribution between the values 0 and 1. The model-predicted probability was calibrated to the actual prevalence level of the HIV/STI in the following manner. First, we ranked the model-predicted probability for each individual in ascending order for the best-selected model. Second, we divided the model testing datasets into 200 probability subgroups. This generated 200 data points for each model-predicted probability and infection prevalence. The choice of 200 was arbitrary but ensured at least 100 individuals were included in each subgroup. Third, we fitted the data using a logistic function to provide a fitting curve for each model-predicted probability and infection prevalence. The calibration process was performed in MATLAB R2019a (details in the Supplementary Materials).

### 2.5. Establishment of the 12-Month HIV/STI Risk-Prediction Tool and Implementation of the Tool on a Web Server

According to the results of the variable importance analysis for all the variables, our final HIV/STI risk-prediction questionnaire was made up of the most important predictors. We used the AUC sensitivity and specificity to re-evaluate the model’s performance. Additionally, we also used the AUC to compare the performance between the best machine learning model using all predictors and the best machine learning model using selected important predictors. Our machine-learning-based risk-prediction tool was developed as a web application using the Shiny R package. Details are in the Section 3 and Supplementary Materials.

## 3. Results

### 3.1. Characteristics of the 12-Month HIV/STI Risk-Prediction Tool Development Data

The proportion of consultations that tested positive over the next 12 months for each infection between 2 March 2015 and 31 December 2019 was: 0.10% (66/65,043) for HIV, 1.32% (750/56,889) for syphilis, 6.70% (4059/60,598) for gonorrhoea, and 7.21% (4578/63,529) for chlamydia. The median age of the individuals was 29.00 (IQR 24.00–43.00) for the four infection datasets (Table 1). Further details are provided in the Supplementary Materials (Table S2).

The incidence was 0.21 [95%CI: 0.17–0.27] per 100 person years (PY) for HIV, 3.42 [95%CI: 3.18–3.67] per 100 PY for syphilis, 17.56 [95%CI: 17.02–18.10] per 100 PY for gonorrhoea, and 18.50 [95%CI: 17.97–19.04] per 100 PY for chlamydia (Table S3). The Kaplan–Meier survival curves for each infection are shown in Figure S1.

### 3.2. Selecting the Best Machine Learning Model for 12-Month HIV/STI Risk-Prediction Tool

Of the 17 models, the receiver operating characteristic (ROC) curve that showed the best prediction models for HIV was the Boosted GLM (AUC = 0.73), for syphilis was the Boosted GLM (AUC = 0.76), for gonorrhoea was the ensemble Elastic-Net Regression (ENR)+ Gradient Boosting Machines (GBM)+ Random Forest (RF) (AUC = 0.73), and for chlamydia was the ensemble ENR+GBM+RF (AUC = 0.67). Details of the model-evaluation metrics are shown in the Tables S4–S19.

### 3.3. Selecting the Most Important Predictors for the 12-Month HIV/STI Risk-Prediction Tool

**Figure 1:**
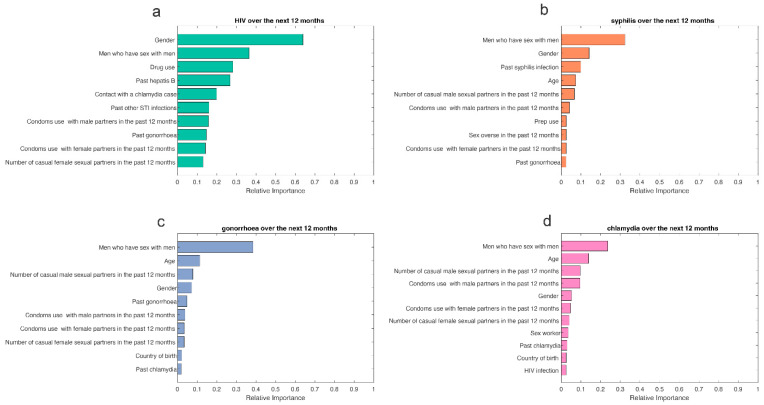
Variable importance analysis for predicting (**a**) HIV, (**b**) syphilis, (**c**) gonorrhoea, and (**d**) chlamydia over the next 12 months.

The results of the variable importance analysis showed the contribution of the predictors for HIV, syphilis, gonorrhoea, and chlamydia acquisition over the next 12 months. The variable importance varies between 0 and 1, with higher values indicating a stronger contribution to the prediction. We used the Boosted GLM variable importance analysis to identify the top predictive factors for HIV and the GBM variable importance analysis for syphilis, gonorrhoea, and chlamydia. Based on the variable importance analyses for HIV, syphilis, gonorrhoea, and chlamydia, the factors that contributed the most to predicting HIV/STIs over the next 12 months included age, gender, sex worker, men who had sex with men in the past 12 months (MSM), country of birth, contact with a chlamydia case, contact with a syphilis case, the number of casual male sexual partners in the past 12 months, condom use with male partners in the past 12 months, condom use with female partners in the past 12 months, drug use, PrEP use, sex overseas in the past 12 months, HIV infection, past chlamydia, past gonorrhoea, past syphilis, past hepatitis B, past genital warts, and past other STIs (Figure 1).

### 3.4. Establishment of the 12-Month HIV/STI Risk-Prediction Model

**Figure 2:**
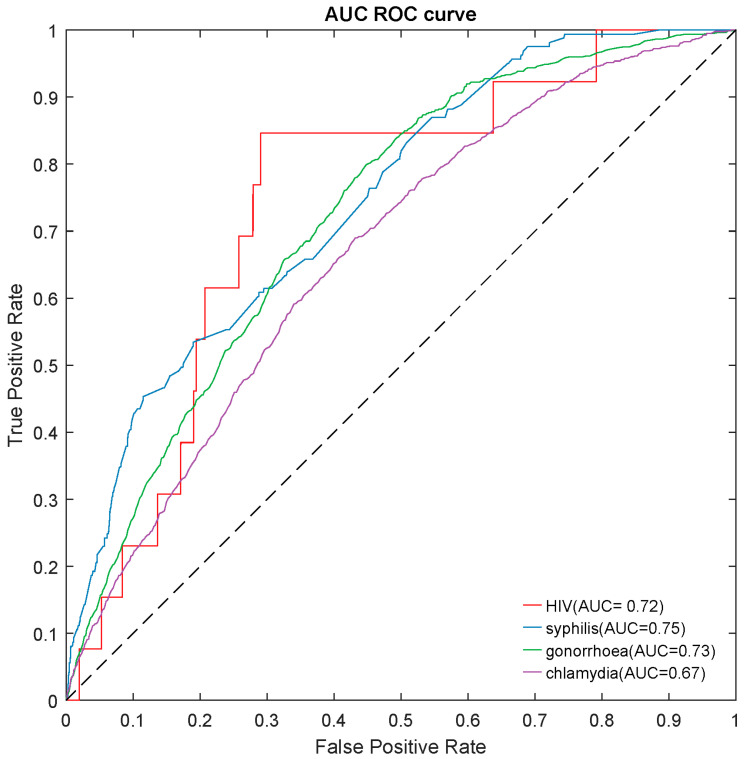
The area under the ROC curve (AUROC) of a risk-prediction tool for predicting HIV/STIs over the next 12 months on testing datasets. STI: syphilis, gonorrhoea, and chlamydia.

We built a risk-prediction model for HIV/STIs over the next 12 months using the most important predictors and the best model. Our risk-prediction model obtained an acceptable performance for predicting HIV (AUC = 0.72), syphilis (AUC = 0.75), gonorrhoea (AUC = 0.73), and chlamydia (AUC = 0.67), similar to its original model based on all the predictors (Figure 2, Tables S20 and S21). Details are shown in the Supplementary Materials.

### 3.5. Twelve-Month HIV/STI Risk Estimates and User Interface

**Figure 3:**
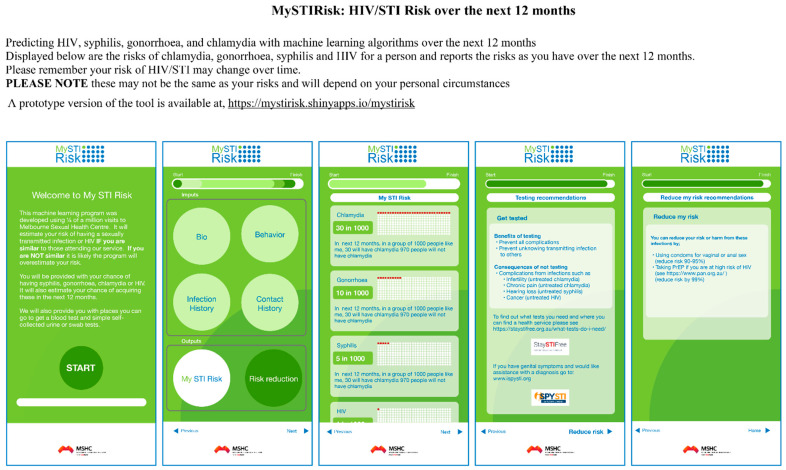
12-month HIV/STI risk-prediction tool’s interface and output. STI: syphilis, gonorrhoea, and chlamydia.

To estimate the risk of twelve-month HIV/STIs, we fitted the data using a logistic function to provide a fitting curve for each model-predicted probability and infection prevalence (see Figures S2–S5). Details are shown in the Supplementary Materials. Our machine learning models were translated into a risk-prediction tool for predicting HIV/STIs. Our machine-learning-based risk-prediction tool was developed as a web application using the Shiny R package that creates the web-based tool named *MySTIRisk*. A prototype version of the tool is available at https://ystirisk.shinyapps.io/mystirisk, accessed on 1 March 2022. Figure 3 shows our proposed design for the user interface. The user interface has five modules: (1) the questionnaire survey module, (2) data-processing module, (3) HIV/STI risk prediction over the next 12 months, (4) testing recommendations, and (5) suggestions for risk reduction (Figure 3). Details are provided in the Supplementary File.

## 4. Discussion

This is the first risk tool we are aware of that uses machine learning algorithms and routinely collects clinical data to predict the risk of acquiring HIV, syphilis, gonorrhoea, and chlamydia over the next 12 months. Our results showed that machine learning techniques could predict the risk of HIV and STIs over the next 12 months with acceptable reliability. Given that this tool uses routinely collected data and provides an immediate future risk, it has a number of potential applications. The potential applications include a web-based program for the public to assess their own future risk or to help clinical services triage high-risk individuals for further frequent screening or early public health interventions. Additional validation in other populations will be needed to evaluate the usefulness of this risk-prediction tool in other countries and regions. Future research should also focus on how best to communicate infection-risk information to the public and use it effectively to encourage them to increase testing or reduce risk and avoid over testing, anxiety, and false reassurance.

Risk prediction tools have been used as a part of interventions in other conditions, including COVID-19 [29,30], cardiovascular diseases [31,32], dementia [33], type 2 diabetes mellitus [34,35], cancer risk [12,36,37], autism [38], and falls [39]. These tools are generally well accepted by users in both public [31,32,33,34,35,36] and health professional domains, although they have mainly been used by health care professionals [30]. The interventions can result in an increased uptake of health information or services, such as screening [11]. For example, a large U.S. cohort used a web-based screening tool and substantially more participants sought information for their mental health [40]. Similarly, a screening app for mental health identified 159 patients from 733 users who were then advised to seek specialised care, of whom 55% started seeing a specialist [41]. Screening risk-assessment tools can also reduce unnecessary screening, as shown by a lung cancer tool that reduced the screening description among those ineligible for screening [37]. The use of apps to assess cardiovascular risk has been advocated as a method of identifying more at-risk individuals who can then access treatment within populations as an ‘add-on’ tool to enhance primary prevention [42]. These authors and others, such as the WHO, comment on the paucity studies investigating the application of risk-assessment tools specifically directed to the public [43,44]. The studies described here highlight the complexity of risk-assessment tools for the public and suggest that improving an individual’s risk perception may lead to better healthcare-seeking behaviour. In a similar vein, previous authors have confirmed that an increase in the risk perception of an STI will likely improve subsequent healthcare use, such as testing or screening [45].

Based on these previous works on risk-assessment tools for other conditions, it is likely that our web-based HIV/STI risk-prediction tool may improve patient care, such as by improving access to sexual health care and increasing uptake and frequency of HIV/STI testing. For example, in California in the United States, a machine learning approach has been used to identify individuals at high risk of HIV and maybe a potential candidate for PrEP [20]. This information could be used to prompt clinicians to customise their intervention for the high-risk population in the clinical setting. However, recent reviews have indicated a relative lack of work relating to the use of AI in promoting HIV testing, and have attributed this lack to limited communication across the many different disciplines that are required for this type of research [46,47]. Individuals who use the tool may increase their HIV/STI risk perception and enable early screening or testing that is essential for HIV/STI prevention and control [48]. Our machine learning models identified some important predictors for HIV/STI acquisition over the next 12 months, consistent with previous studies. Previous research found various factors related to a high risk of incident HIV/STI, such as MSM [49], age (older for HIV and younger for STIs) [50], symptoms of or previous STIs [51], inconsistent condom use, PrEP use, and injecting drug use [52]. Providing our risk-prediction tool to these high-risk populations may improve the HIV/STI testing rate. However, we are also aware of the potential risk that an inappropriate interpretation of the risk score may lead some high-risk individuals to reduce their testing or some low-risk individuals to possibly test inappropriately. It is also possible that the tool may lead to an increase in anxiety about HIV/STIs in some individuals. However, even for cancer risk assessment, this concern was relatively minor for the case of breast cancer risk prediction [12].

Our web-based HIV/STI risk-prediction tool may offer a useful method for potential sexual behavioural interventions to reduce future HIV/STI risk in addition to just promoting testing [53]. An example of this exists in cardiovascular risk where researchers used an individual’s risk as part of an intervention for better lifestyle behaviours, including reducing smoking, more exercise, improving nutrition, and less stress [54]. The intervention group in this trial was shown to have more than two times a reduction in the Framingham scores for cardiovascular diseases than the control group over the next 12 months [54]. Therefore, in addition to potentially increasing testing, our HIV/STI risk-prediction tool could be incorporated into other preventive interventions, such as using PrEP. Such an addition would address one of the major challenges to increasing the PrEP update, which is identifying individuals who may benefit from HIV PrEP [55]. Nevertheless, we are aware of the possibility that providing risk scores and suggestions may not significantly change the targeted behaviour, as demonstrated by a randomised controlled trial study on cancer risk [56]. We recommend further controlled studies to examine if our HIV/STI risk-prediction tool would alter both short- and long-term behaviours.

This study has several limitations. First, the validity of the results depends on the accuracy of the self-reported information, which is subject to recall, non-response, and the social-desirability bias. Substantial work has been undertaken on our computer-assisted self-interviewing (CASI) system’s validity and accuracy to ensure it performs well [57]. Second, the biggest challenge in developing our HIV risk-prediction model was the low incidence of HIV [19]. The HIV dataset had highly imbalanced data, with only 0.1% of the consultations having a positive HIV result. To address the problem of the limited HIV-positive samples in our machine learning training models, future machine learning models may employ more sophisticated machine learning techniques (e.g., transfer learning) [58], which may improve the accuracy of the models. In addition, the HIV data included a very small proportion of females, so our findings may not be generalisable to female users. Third, we used data from one clinic that services a population with a specific incidence of infection and demographic characteristics. This may not be representative of other population groups in the country or other global settings. Therefore, if users accessing the tool are not similar to those attending our clinical services, the risk estimate may be incorrect. However, one study comparing our clinic attendees and users accessing the MSHC website demonstrated similar characteristics and behaviour [59]. Further validation will be required if the prediction tool is used in other countries and regions. Fourth, the risks of HIV have changed rapidly over time and may continue to change. For example, the introduction of PrEP reduced HIV risk substantially, but condom use declined in the pre-exposure prophylaxis era [6]. Fifth, our models did not include data among individuals who tested positive on the day they conducted their questionnaire. This means that our estimated risk may be lower than it would have otherwise been. We did so to ensure that we measured the incidence of HIV/STI correctly. Sixth, the tool may be further improved by including more detailed behavioural information. For example, kissing and sequential sexual practices may contribute to gonorrhoea infection at more than one anatomical site [60]. Future HIV/STI predictive models may include these factors to improve the model’s accuracy.

## 5. Conclusions

Our study demonstrates that EHR-based machine learning can predict HIV/STIs over the next 12 months. Based on the EHR in one of Australia’s largest sexual health clinics, our web-based risk-assessment tool has an acceptable reliability in predicting the risk of HIV and three recurrent and asymptomatic STIs over the next 12 months. The risk-assessment tool can also be incorporated into a clinic to promote future HIV/STI testing or identify individuals for HIV pre-exposure prophylaxis or early interventions for the reduction in future HIV/STI risk. Further validation studies in other countries can assess the usefulness of this risk-assessment tool, which helps reduce HIV/STI incidence and the cost of HIV/STI screening that requires expensive equipment and specialised expertise.

## Figures and Tables

**Table 1:** Characteristics (proportion or median value) of the included subjects stratified by HIV and STIs over the next 12 months.

Predictors	HIV		Syphilis		Gonorrhoea		Chlamydia	
	No	Yes	No	Yes	No	Yes	No	Yes
	*n* (%)	*n* (%)	*n* (%)	*n* (%)	*n* (%)	*n* (%)	*n* (%)	*n* (%)
Gender								
Female	16,478 (25.4%)	1 (1.5%)	14,476 (25.8%)	12 (1.6%)	18,018 (31.9%)	298 (7.3%)	18,652 (31.6%)	687 (15.0%)
Male	48,499 (74.6%)	65 (98.5%)	41,663 (74.2%)	738 (98.4%)	38,521 (68.1%)	3761 (92.7%)	40,299 (68.4%)	3891 (85.0%)
Men who have sex with men								
No	5797 (12.0%)	1 (1.5%)	3854 (9.3%)	14 (1.9%)	5036 (13.1%)	55 (1.5%)	6713 (16.7%)	403 (10.4%)
Yes	42,702 (88.0%)	64 (98.5%)	37,809 (90.7%)	724 (98.1%)	33,485 (86.9%)	3706 (98.5%)	33,586 (83.3%)	3488 (89.6%)
Country of birth								
Australia	30,473 (46.9%)	29 (43.9%)	25,887 (46.1%)	355 (47.3%)	25,587 (45.3%)	2023 (49.8%)	27,081 (45.9%)	2112 (46.1%)
Overseas	31,978 (49.2%)	34 (51.5%)	28,099 (50.1%)	367 (48.9%)	28812 (51.0%)	1900 (46.8%)	29,684 (50.4%)	2310 (50.5%)
Missing	2526 (3.9%)	3 (4.5%)	2153 (3.8%)	28 (3.7%)	2140 (3.8%)	136 (3.4%)	2186 (3.7%)	156 (3.4%)
Age at consultation								
Median [IQR]	29.0 (25.0, 35.0)	30.5 (27.0, 43.0)	29.0 (25.0, 36.0)	30.0 (26.0, 37.0)	29.0 (25.0, 35.0)	29.0 (25.0, 34.0)	29.0 (25.0, 35.0)	28.0 (24.0, 34.0)
Current PrEP use								
No	62,195 (95.7%)	64 (97.0%)	53,496 (95.3%)	658 (87.7%)	53,998 (95.5%)	3656 (90.1%)	56,519 (95.9%)	4167 (91.0%)
Yes	2782 (4.3%)	2 (3.0%)	2643 (4.7%)	92 (12.3%)	2541 (4.5%)	403 (9.9%)	2432 (4.1%)	411 (9.0%)
Current sex worker								
No	57,383 (88.3%)	65 (98.5%)	49,068 (87.4%)	736 (98.1%)	49,458 (87.5%)	3902 (96.1%)	51,981 (88.2%)	4418 (96.5%)
Yes	7594 (11.7%)	1 (1.5%)	7071 (12.6%)	14 (1.9%)	7081 (12.5%)	157 (3.9%)	6970 (11.8%)	160 (3.5%)

Note: IQR: interquartile range.

## Data Availability

The data is not publicly available due to privacy or ethical restrictions but will be made available on reasonable request from the corresponding author, with the permission of the Alfred Hospital Ethics Committee. Restrictions apply to the availability of the data used under the license for this study.

## Supplementary Materials

The following supporting information can be downloaded at: https://www.mdpi.com/article/10.3390/jcm11071818/s1, Figure S1. The estimated survival curves for HIV and sexually transmitted infections using Kaplan-Meier. sexually transmitted infections: syphilis, gonorrhoea, and chlamydia. Kaplan-Meier survival curves stratified by risk groups over 365 days. Figure S2. The fitting curve of model-predicted probability and HIV prevalence over the next 12 months. Figure S3. The fitting curve of model-predicted probability and syphilis prevalence over the next 12 months. Figure S4. The fitting curve of model-predicted probability and gonorrhoea prevalence over the next 12 months. Figure S5. The fitting curve of model-predicted probability and chlamydia prevalence over the next 12 months. Table S1. (a) Inclusion and exclusion of HIV data. (b) Inclusion and exclusion of syphilis, gonorrhoea, and chlamydia data. Table S2. Characteristics (proportion or median value) of the included subjects stratified by HIV and STIs over the next 12 months. STIs: sexually transmitted infections. Table S3. Incidence of HIV and sexually transmitted infections per 100 person-years with 95% confidence intervals. sexually transmitted infections: syphilis, gonorrhoea, and chlamydia. Table S4. The area under ROC curve (AUC) of all predictors for predicting HIV over the next 12 months on testing data. ROC curve: receiver operating characteristic curve. Table S5. The area under ROC curve (AUC) of all predictors for predicting syphilis over the next 12 months on testing data. ROC curve: receiver operating characteristic curve. Table S6. The area under ROC curve (AUC) of all predictors for predicting gonorrhoea over the next 12 months on testing data. ROC curve: receiver operating characteristic curve. Table S7. The area under ROC curve (AUC) of all predictors for predicting chlamydia over the next 12 months on testing data. ROC curve: receiver operating characteristic curve. Table S8. Sensitivity of all predictors for predicting HIV over the next 12 months on testing data. Table S9. Sensitivity of all predictors for predicting syphilis over the next 12 months on testing data. Table S10. Sensitivity of all predictors for predicting gonorrhoea over the next 12 months on testing data. Table S11. Sensitivity of all predictors for predicting chlamydia over the next 12 months on testing data. Table S12. Specificity of all predictors for predicting HIV over the next 12 months on testing data. Table S13. Specificity of all predictors for predicting syphilis over the next 12 months on testing data. Table S14. Specificity of all predictors for predicting gonorrhoea over the next 12 months on testing data. Table S15. Specificity of all predictors for predicting chlamydia over the next 12 months on testing data. Table S16. F1 of all predictors for predicting HIV over the next 12 months on testing data. Table S17. F1 of all predictors for predicting syphilis over the next 12 months on testing data. Table S18. F1 of all predictors for predicting gonorrhoea over the next 12 months on testing data. Table S19. F1 of all predictors for predicting chlamydia over the next 12 months on testing data. Table S20. Performance metrics of the 12-month HIV/STI risk prediction tool (Best machine learning models using selected predictors). STI: syphilis, gonorrhoea, and chlamydia. Table S21. The performance comparison of the best machine learning models using all predictors and risk prediction tool. References [26,27,28,50,61] are cited in the Supplementary Materials.
