# Supplementary material for: A Machine-Learning-Based Risk-Prediction Tool for HIV and Sexually Transmitted Infections Acquisition over the Next 12 Months
Source: J Clin Med. 2022 Mar 25;11(7):1818. doi: 10.3390/jcm11071818 (PMC8999359; doi:10.3390/jcm11071818)
Supplement: Supplementary file 1 [file jcm-11-01818-s001.zip › jcm-1642000-supplementary.pdf]

Supplementary File: A Machine Learning-Based Risk Prediction Tool for HIV and Sexually Transmitted Infections Acquisition over the next 12 Months

Contents

Supplementary Methods 3

Data cleaning procedure 3

Table S1. (a) Inclusion and exclusion of HIV data. (**b**) Inclusion and exclusion of syphilis, gonorrhoea, and chlamydia data. 4

Machine learning training techniques 7

Supplementary Results 8

Table S2. Characteristics (proportion or median value) of the included subjects stratified by HIV and STIs over the next 12 months 8

Figure S1. The estimated survival curves for HIV and sexually transmitted infections using Kaplan-Meier 17

Table S3. Incidence of HIV and sexually transmitted infections per 100 person-years with 95% confidence intervals 18

Development of machine learning models using all predictors 19

Table S4. The area under ROC curve (AUC) of all predictors for predicting HIV over the next 12 months on testing data 19

Table S5. The area under ROC curve (AUC) of all predictors for predicting syphilis over the next 12 months on testing data 20

Table S6. The area under ROC curve (AUC) of all predictors for predicting gonorrhoea over the next 12 months on testing data 22

Table S7. The area under ROC curve (AUC) of all predictors for predicting chlamydia over the next 12 months on testing data 23

Table S8. Sensitivity of all predictors for predicting HIV over the next 12 months on testing data.. 25

Table S9. Sensitivity of all predictors for predicting syphilis over the next 12 months on testing data 27

Table S10. Sensitivity of all predictors for predicting gonorrhoea over the next 12 months on testing data 28

Table S11. Sensitivity of all predictors for predicting chlamydia over the next 12 months on testing data 30

Table S12. Specificity of all predictors for predicting HIV over the next 12 months on testing data 31

Table S13. Specificity of all predictors for predicting syphilis over the next 12 months on testing data 33

Table S14. Specificity of all predictors for predicting gonorrhoea over the next 12 months on testing data 34

Table S15. Specificity of all predictors for predicting chlamydia over the next 12 months on testing data 36

Table S16. F1 of all predictors for predicting HIV over the next 12 months on testing data 37

Table S17. F1 of all predictors for predicting syphilis over the next 12 months on testing data 39

Table S18. F1 of all predictors for predicting gonorrhoea over the next 12 months on testing data . 40

Table S19. F1 of all predictors for predicting chlamydia over the next 12 months on testing data ... 42

Performance metrics of the 12-month HIV/STI risk prediction tool 44

Table S20. Performance metrics of the 12-month HIV/STI risk prediction tool (Best machine learning models using selected predictors) 44

Table S21. The performance comparison of the best machine learning models using all predictors and risk prediction tool 45

12-month HIV/STI risk estimate 47

Figure S2. The fitting curve of model-predicted probability and HIV prevalence over the next 12 months. 47

Figure S3. The fitting curve of model-predicted probability and syphilis prevalence over the next 12 months. 48

Figure S4. The fitting curve of model-predicted probability and gonorrhoea prevalence over the next 12 months. 49

Figure S5. The fitting curve of model-predicted probability and chlamydia prevalence over the next 12 months. 50

User interface design 51

Reference 52

Supplementary Methods

Data cleaning procedure

This study defined the "visit interval" as the period between two consecutive testing visits. Therefore, if the same individual attended many times over the study period, they would have more than one "visit interval". We also defined the 'first test' as the first test of the two consecutive tests.

Only individuals with at least two HIV tests at least 30 days apart were included. Once an individual tested positive, all subsequent data on HIV testing were excluded [1]. If individuals had negative HIV tests more than 12 months after a negative test, assuming they were negative within 12 months. If the time between an initial negative test and subsequent positive tests was between 12 months and 13 months, the interval was re-assigned to 12 months. We assumed that the infection occurred halfway between the two tests for those where the interval was between 13 months and 24 months. The midpoint of the latest-negative and earliest positive test date can be used to infer the time of the infection event [2]. If the time was greater than 24 months, the data was excluded. For HIV analysis, detailed inclusions and exclusions are in Supplementary Table S1a and Supplementary Figure S1.

Data were included if an individual had at least two tests for the infection under analysis. We excluded the testing intervals of more than 13 months because the natural history will have resulted in some cures without treatment. We also excluded individuals who tested positive on their initial tests because we planned to use this data to develop a web-based tool. Individuals using this tool would not test on the day, and so any future tests may include prevalent infections they had on the day they did the web-based tool. This would mean that if they went for testing soon after using the tool, their estimate would be too high because it included prevalent infections. By excluding positive cases, we acknowledge that their estimated risk will be underestimated. Our analysis included

65,043 consultations that had testing for HIV, 56,889 consultations for syphilis, 60,598 consultations for gonorrhoea and 63,529 consultations for chlamydia. For syphilis, gonorrhoea, and chlamydia analysis, detailed inclusions and exclusions are in Supplementary Table S1a,b and Supplementary Figure S1.

Table S1. **(a)** Inclusion and exclusion of HIV data. (**b**) Inclusion and exclusion of syphilis, gonorrhoea, and chlamydia data.

**(a)**								
**HIV**								
Simulated ID number		Visit 1	Visit 2	Visit 3	Visit 4	Visit 5	Visit 6	Visit 7
2	Diagnosis	negative	negative	negative	positive	positive	positive	positive
	Visit interval between two consecutive dates		Visit interval between visit 2 and visit 1	Visit interval between visit 3 and visit 2	Visit interval between visit 4 and visit 3	Visit interval between visit 5 and visit 4	Visit interval between visit 6 and visit 5	Visit interval between visit 7 and visit 6
	Eligible (yes/no)	---	Eligible	eligible	eligible	Not eligible	Not eligible	Not eligible
Data cleaning, eligible data								
Simulated ID number	Period data	Current behaviour data	Future diagnosis outcome data	Process of progression				

2	Visit interval between visit 2 and visit 1	behaviour data on Visit 1 date	diagnosis on Visit 2 date (negative)	from negative to negative				
2	Visit interval between visit 3 and visit 2	behaviour data on Visit 2 date	diagnosis on Visit 3 date (negative)	from negative to negative				
2	Visit interval between visit 4 and visit 3	behaviour data on Visit 3 date	diagnosis on Visit 4 date (positive)	from negative to positive				

Note:

1. Include from 1) ‘first’ negative to negative for any two consecutive diagnoses or 2) from ‘first’ negative to positive and 3) exclude all data after a positive diagnosis for the same individual
2. We assumed that the patient must be negative within 12 months for those who were always negative diagnoses.
3. For those who progressed from negative to positive, and visit interval was between 12 months and 13 months, and then the revisit interval was re-assigned to 12 months because of the delay in follow-up.
4. For those who progressed from negative to positive, the visit interval was between 13 months and 24 months, and we assumed that the infection occurred in the middle time. For example, if the revisit interval was 16 months, then the middle time was 8 months. We divided the revisit interval by two to get the final revisit interval.
5. After the above data processing, the revisit interval will not exceed 12 months.

(b)

**NG-CT-syphilis data**								
Simulated ID		Visit 1	Visit 2	Visit 3	Visit 4	Visit 5	Visit 6	Visit 6

number								
1	Diagnosis	negative	positive	positive	negative	positive	negative	negative
	Visit interval between two consecutive visits		Visit interval between visit 2 and visit 1	Visit interval between visit 3 and visit 2	Visit interval between visit 4 and visit 3	Visit interval between visit 5 and visit 4	Visit interval between visit 6 and visit 5	Visit interval between visit 7 and visit 6
	**Eligible (Yes/ No)**		Eligible	Not eligible	Not eligible	Eligible	Not eligible	Eligible
**After data cleaning**								
Simulated ID number	Period Data	Current behaviour data	Future diagnosis outcome data	Process of progression				
1	Visit interval 2-1	behaviour data on visit 1 date	diagnosis on visit 2 date (positive)	from negative to positive				
	Visit interval 5-4	behaviour data on visit 4 date	diagnosis on visit 5 date (positive)	from negative to positive				

	Visit interval 7-6	behaviour data on visit 6 date	diagnosis on visit 7 date (negative)	from negative to negative				

Note:

1. We included patients who had at least two tests within 13 months and excluded visit intervals> 13 months.
2. Transgender and patients aged below 18 years were excluded.
3. If the revisit interval was between 12 months and 13 months, then the revisit interval was re-assigned to 12 months because of the delay in follow-up.
4. **Include from** 1) 'first' negative to negative for any two consecutive diagnoses or 2) from 'first' negative to positive for any two consecutive diagnoses, and exclude from 3) 'first' positive to negative or 4) from 'first' positive to positive for any two consecutive diagnoses
5. After the above data processing, the revisit interval will not exceed 12 months.

Machine learning training techniques

For STI models, we used the nested cross-validation (five outer folds, ten inner folds) method to better estimate the generalisation error and solve the overfitting and selection bias caused by using a single data set for the model selection and model training [3]. The external cross-validation loop was repeated five times to solve variance caused by the choice of data set to split. The prevalence of each of the four infections was below 10%, which means the data is imbalanced. Imbalanced data may cause either over-fitted or under-performed prediction results [4]. We used random under-sampling in the training dataset to address data imbalance to solve the class

imbalance problem. Furthermore, an inner 10-fold CV loop was created for each model to select the tuning hyper-parameters for the maximised area under the ROC curve (AUC) on the training fold [5]. For HIV models, we used an 80:20 random under-sampling split based on outcome (HIV infection status) to create training dataset and testing dataset for analysis due to only 0.1% of consultations had a positive HIV result. All HIV models were trained using the training dataset with a ten-fold cross-validation method and assessed the model performance on the testing dataset.

Supplementary Results

Table S2. Characteristics (proportion or median value) of the included subjects stratified by HIV and STIs over the next 12 months.

**Predictors**	**HIV**		**syphilis**		**gonorrhoea**		**chlamydia**	
	(No)	(Yes)	(No)	(Yes)	(No)	(Yes)	(No)	(Yes)
**In the last 12 months, have you had sex with a male**								
No	10479 (16.1%)	3 (4.5%)	8079 (14.4%)	40 (5.3%)	9276 (16.4%)	168 (4.1%)	11059 (18.8%)	547 (11.9%)
Yes	52089 (80.2%)	61 (92.4%)	45725 (81.4%)	665 (88.7%)	44988 (79.6%)	3702 (91.2%)	45648 (77.4%)	3843 (83.9%)
Unknown/ Missing	2409 (3.7%)	2 (3.0%)	2335 (4.2%)	45 (6.0%)	2275 (4.0%)	189 (4.7%)	2244 (3.8%)	188 (4.1%)
**Condoms use with male partners in the past 12 months**								

Always	16133 (24.8%)	14 (21.2%)	14259 (25.4%)	138 (18.4%)	13298 (23.5%)	963 (23.7%)	13691 (23.2%)	843 (18.4%)
Never	4000 (6.2%)	4 (6.1%)	3418 (6.1%)	60 (8.0%)	3598 (6.4%)	252 (6.2%)	3569 (6.1%)	269 (5.9%)
Not applicable (no vag/anal sex)	1064 (1.6%)	1 (1.5%)	901 (1.6%)	6 (0.8%)	912 (1.6%)	36 (0.9%)	934 (1.6%)	29 (0.6%)
Unknown or Declined to answer	2798 (4.3%)	2 (3.0%)	2692 (4.8%)	61 (8.1%)	2659 (4.7%)	208 (5.1%)	2648 (4.5%)	223 (4.9%)
Usually (>50%) or sometimes	29453 (45.3%)	40 (60.6%)	25859 (46.1%)	432 (57.6%)	25954 (45.9%)	2352 (57.9%)	26189 (44.4%)	2613 (57.1%)
Missing	11529 (17.7%)	5 (7.6%)	9010 (16.0%)	53 (7.1%)	10118 (17.9%)	248 (6.1%)	11920 (20.2%)	601 (13.1%)
**In the last 12 months, have you had sex with a female**								
No	49620 (76.4%)	57 (86.4%)	43930 (78.3%)	625 (83.3%)	43220 (76.4%)	3468 (85.4%)	43615 (74.0%)	3641 (79.5%)
Yes	13018 (20.0%)	5 (7.6%)	9943 (17.7%)	85 (11.3%)	11088 (19.6%)	395 (9.7%)	13148 (22.3%)	743 (16.2%)
Unknown/ Missing	2339 (3.6%)	4 (6.1%)	2266 (4.0%)	40 (5.3%)	2231 (3.9%)	196 (4.8%)	2188 (3.7%)	194 (4.2%)
**Condoms use with female partners in the past 12 months**								

Always	2390 (3.7%)	2 (3.0%)	1871 (3.3%)	17 (2.3%)	1729 (3.1%)	91 (2.2%)	2111 (3.6%)	97 (2.1%)
Never	1273 (2.0%)	0 (0%)	1015 (1.8%)	13 (1.7%)	1078 (1.9%)	44 (1.1%)	1262 (2.1%)	75 (1.6%)
Not applicable (no vag/anal sex)	6170 (9.5%)	6 (9.1%)	5677 (10.1%)	97 (12.9%)	5275 (9.3%)	516 (12.7%)	5288 (9.0%)	493 (10.8%)
Unknown or Declined to answer	1458 (2.2%)	2 (3.0%)	1410 (2.5%)	48 (6.4%)	1379 (2.4%)	160 (3.9%)	1355 (2.3%)	178 (3.9%)
Usually (>50%) or Sometimes	6681 (10.3%)	3 (4.5%)	4739 (8.4%)	54 (7.2%)	5659 (10.0%)	194 (4.8%)	6993 (11.9%)	487 (10.6%)
Missing	47005 (72.3%)	53 (80.3%)	41427 (73.8%)	521 (69.5%)	41419 (73.3%)	3054 (75.2%)	41942 (71.1%)	3248 (70.9%)
**When was the last time you injected drugs**								
Less than 12 months ago	287 (0.4%)	1 (1.5%)	248 (0.4%)	10 (1.3%)	248 (0.4%)	30 (0.7%)	250 (0.4%)	31 (0.7%)
Less than 3 months ago	693 (1.1%)	5 (7.6%)	562 (1.0%)	43 (5.7%)	526 (0.9%)	101 (2.5%)	551 (0.9%)	88 (1.9%)
More than 12 months ago	696 (1.1%)	1 (1.5%)	616 (1.1%)	11 (1.5%)	614 (1.1%)	45 (1.1%)	626 (1.1%)	47 (1.0%)
Never injected	60910 (93.7%)	55 (83.3%)	52384 (93.3%)	628 (83.7%)	52915 (93.6%)	3651 (89.9%)	55275 (93.8%)	4188 (91.5%)
Missing	2391 (3.7%)	4 (6.1%)	2329 (4.1%)	58 (7.7%)	2236 (4.0%)	232 (5.7%)	2249 (3.8%)	224 (4.9%)

**Sex oversea (outside Australia or New Zealand) in the last 12 months**								
No	37796 (58.2%)	39 (59.1%)	32593 (58.1%)	418 (55.7%)	32689 (57.8%)	2220 (54.7%)	33903 (57.5%)	2466 (53.9%)
Yes	20072 (30.9%)	20 (30.3%)	17053 (30.4%)	206 (27.5%)	17660 (31.2%)	1272 (31.3%)	18738 (31.8%)	1564 (34.2%)
Missing	7109 (10.9%)	7 (10.6%)	6493 (11.6%)	126 (16.8%)	6190 (10.9%)	567 (14.0%)	6310 (10.7%)	548 (12.0%)
**Contact with gonorrhea**								
No/Missing	63256 (97.4%)	63 (95.5%)	54563 (97.2%)	716 (95.5%)	55282 (97.8%)	3842 (94.7%)	57481 (97.5%)	4352 (95.1%)
Yes	1721 (2.6%)	3 (4.5%)	1576 (2.8%)	34 (4.5%)	1257 (2.2%)	217 (5.3%)	1470 (2.5%)	226 (4.9%)
**Contact with chlamydia**								
No/Missing	63392 (97.6%)	61 (92.4%)	54774 (97.6%)	724 (96.5%)	54873 (97.1%)	3892 (95.9%)	57573 (97.7%)	4399 (96.1%)
Yes	1585 (2.4%)	5 (7.6%)	1365 (2.4%)	26 (3.5%)	1666 (2.9%)	167 (4.1%)	1378 (2.3%)	179 (3.9%)
**Contact with syphilis**								
No/Missing	64306 (99.0%)	63 (95.5%)	55533 (98.9%)	726 (96.8%)	56039 (99.1%)	3955 (97.4%)	58418 (99.1%)	4510 (98.5%)
Yes	671 (1.0%)	3 (4.5%)	606 (1.1%)	24 (3.2%)	500 (0.9%)	104 (2.6%)	533 (0.9%)	68 (1.5%)

**STI symptoms**								
No	41779 (64.3%)	39 (59.1%)	36126 (64.4%)	440 (58.7%)	34278 (60.6%)	2504 (61.7%)	36396 (61.7%)	2685 (58.7%)
Yes	15737 (24.2%)	22 (33.3%)	13162 (23.4%)	224 (29.9%)	15961 (28.2%)	1029 (25.4%)	16362 (27.8%)	1361 (29.7%)
Missing	7461 (11.5%)	5 (7.6%)	6851 (12.2%)	86 (11.5%)	6300 (11.1%)	526 (13.0%)	6193 (10.5%)	532 (11.6%)
**Past genital warts infection ever**								
No	21309 (32.8%)	37 (56.1%)	18715 (33.3%)	390 (52.0%)	19030 (33.7%)	1850 (45.6%)	19576 (33.2%)	1994 (43.6%)
Yes	4337 (6.7%)	6 (9.1%)	3849 (6.9%)	57 (7.6%)	3778 (6.7%)	373 (9.2%)	4013 (6.8%)	381 (8.3%)
Missing	39331 (60.5%)	23 (34.8%)	33575 (59.8%)	303 (40.4%)	33731 (59.7%)	1836 (45.2%)	35362 (60.0%)	2203 (48.1%)
**Past chlamydia infection ever**								
No	10873 (16.7%)	19 (28.8%)	9492 (16.9%)	180 (24.0%)	9092 (16.1%)	880 (21.7%)	9545 (16.2%)	823 (18.0%)
Yes	14773 (22.7%)	24 (36.4%)	13072 (23.3%)	267 (35.6%)	13716 (24.3%)	1343 (33.1%)	14044 (23.8%)	1552 (33.9%)
Missing	39331 (60.5%)	23 (34.8%)	33575 (59.8%)	303 (40.4%)	33731 (59.7%)	1836 (45.2%)	35362 (60.0%)	2203 (48.1%)

**Past gonorrhea infection ever**								
No	13650 (21.0%)	14 (21.2%)	11700 (20.8%)	170 (22.7%)	13028 (23.0%)	775 (19.1%)	13637 (23.1%)	1108 (24.2%)
Yes	11996 (18.5%)	29 (43.9%)	10864 (19.4%)	277 (36.9%)	9780 (17.3%)	1448 (35.7%)	9952 (16.9%)	1267 (27.7%)
Missing	39331 (60.5%)	23 (34.8%)	33575 (59.8%)	303 (40.4%)	33731 (59.7%)	1836 (45.2%)	35362 (60.0%)	2203 (48.1%)
**Past NSU infection**								
No	20834 (32.1%)	42 (63.6%)	18367 (32.7%)	425 (56.7%)	17141 (30.3%)	2061 (50.8%)	17653 (29.9%)	2073 (45.3%)
Yes	757 (1.2%)	1 (1.5%)	669 (1.2%)	16 (2.1%)	683 (1.2%)	72 (1.8%)	690 (1.2%)	66 (1.4%)
Missing	43386 (66.8%)	23 (34.8%)	37103 (66.1%)	309 (41.2%)	38715 (68.5%)	1926 (47.5%)	40608 (68.9%)	2439 (53.3%)
**Past syphilis infection**								
No	21366 (32.9%)	32 (48.5%)	18739 (33.4%)	252 (33.6%)	19254 (34.1%)	1655 (40.8%)	20101 (34.1%)	1831 (40.0%)
Yes	4280 (6.6%)	11 (16.7%)	3825 (6.8%)	195 (26.0%)	3554 (6.3%)	568 (14.0%)	3488 (5.9%)	544 (11.9%)
Missing	39331 (60.5%)	23 (34.8%)	33575 (59.8%)	303 (40.4%)	33731 (59.7%)	1836 (45.2%)	35362 (60.0%)	2203 (48.1%)
**Past genital herpes infection**								

No	24312 (37.4%)	44 (66.7%)	21382 (38.1%)	452 (60.3%)	21531 (38.1%)	2152 (53.0%)	22254 (37.8%)	2287 (50.0%)
Yes	2687 (4.1%)	2 (3.0%)	2400 (4.3%)	36 (4.8%)	2499 (4.4%)	180 (4.4%)	2525 (4.3%)	229 (5.0%)
Missing	37978 (58.4%)	20 (30.3%)	32357 (57.6%)	262 (34.9%)	32509 (57.5%)	1727 (42.5%)	34172 (58.0%)	2062 (45.0%)
**HIV infection**	-	-						
No	-	-	21864 (38.9%)	394 (52.5%)	22002 (38.9%)	2031 (50.0%)	22812 (38.7%)	2198 (48.0%)
Yes	-	-	700 (1.2%)	53 (7.1%)	806 (1.4%)	192 (4.7%)	777 (1.3%)	177 (3.9%)
Missing	-	-	33575 (59.8%)	303 (40.4%)	33731 (59.7%)	1836 (45.2%)	35362 (60.0%)	2203 (48.1%)
**Past Hepatitis B infection**								
No	25324 (39.0%)	43 (65.2%)	22267 (39.7%)	439 (58.5%)	22525 (39.8%)	2197 (54.1%)	23310 (39.5%)	2342 (51.2%)
Yes	322 (0.5%)	0 (0%)	297 (0.5%)	8 (1.1%)	283 (0.5%)	26 (0.6%)	279 (0.5%)	33 (0.7%)
Missing	39331 (60.5%)	23 (34.8%)	33575 (59.8%)	303 (40.4%)	33731 (59.7%)	1836 (45.2%)	35362 (60.0%)	2203 (48.1%)
**Past Hepatitis C infection**								
No	25511 (39.3%)	41 (62.1%)	22442 (40.0%)	437 (58.3%)	22673 (40.1%)	2207 (54.4%)	23452 (39.8%)	2359 (51.5%)
Yes	135 (0.2%)	2 (3.0%)	122 (0.2%)	10 (1.3%)	135 (0.2%)	16 (0.4%)	137 (0.2%)	16 (0.3%)

Missing	39331 (60.5%)	23 (34.8%)	33575 (59.8%)	303 (40.4%)	33731 (59.7%)	1836 (45.2%)	35362 (60.0%)	2203 (48.1%)
**Other past STI infections**								
No	25511 (39.3%)	41 (62.1%)	22442 (40.0%)	437 (58.3%)	22673 (40.1%)	2207 (54.4%)	23452 (39.8%)	2359 (51.5%)
Yes	135 (0.2%)	2 (3.0%)	122 (0.2%)	10 (1.3%)	135 (0.2%)	16 (0.4%)	137 (0.2%)	16 (0.3%)
Missing	39331 (60.5%)	23 (34.8%)	33575 (59.8%)	303 (40.4%)	33731 (59.7%)	1836 (45.2%)	35362 (60.0%)	2203 (48.1%)
**In the past 12 months, how many men have you had sex with**								
Median [IQR]	5.00 [3.0, 10.0]	10.0 [4.0, 15.5]	5.00 [3.0, 10.0]	7.00 [4.0, 20.0]	5.00 [3.0, 10.0]	8.00 [4.0, 16.0]	5.00 [3.0, 10.0]	6.00 [4.0, 15.0]
**In the past 12 months, how many women have you had sex with**								
Median [IQR]	3.00 [2.0, 6.0]	4.00 [1.8, 6.8]	3.00 [1.0, 6.0]	2.00 [1.00, 5.0]	3.00 [2.0, 6.0]	2.00 [1.00, 5.0]	3.00 [2.00, 6.0]	4.00 [2.00, 8.0]

Note: IQR is the first and third interquartile range.

The survival curves for HIV and STIs using Kaplan-Meier

We used the Kaplan-Meier approach for plotting survival curves. We developed survival curves to show the proportion of individuals who remained negative for each infection within 12 months. There were 99.68% (95%CI: 99.59%-99.76%) for HIV, 93.00% (95%CI: 92.11%-93.91%) for syphilis, 73.08% (95%CI: 71.67%-74.52%) for gonorrhoea, 70.65% (95%CI: 69.30%-72.03%) for chlamydia.

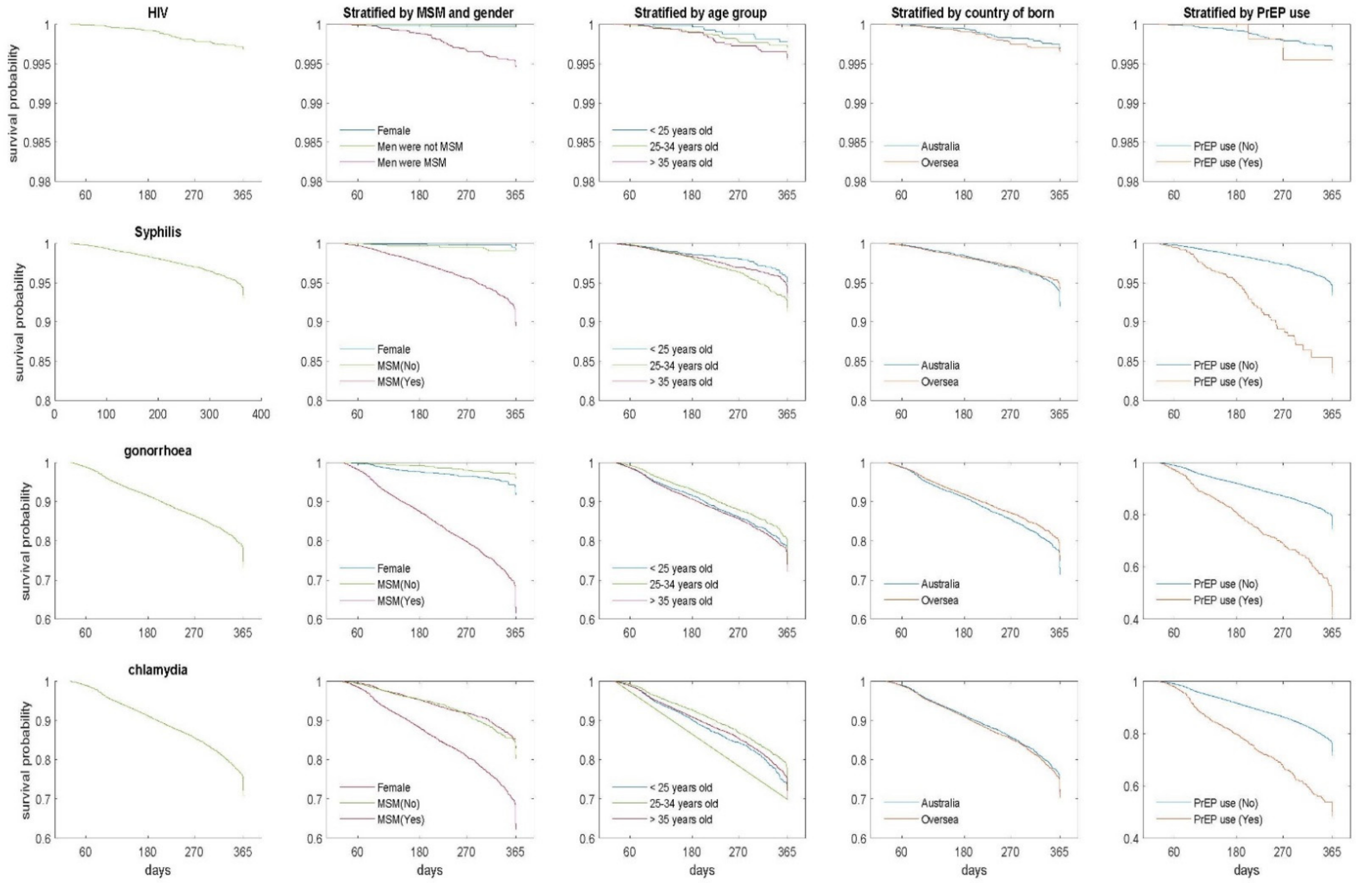

Figure S1. The estimated survival curves for HIV and sexually transmitted infections using Kaplan-Meier.

Table S3. Incidence of HIV and sexually transmitted infections per 100 person-years with 95% confidence intervals.

	**HIV**<br><br>**mean/ 95%CI**	**syphilis**<br><br>**mean/ 95%CI**	**gonorrhoea**<br><br>**mean/ 95%CI**	**chlamydia**<br><br>**mean/ 95%CI**
**Overall**	0.214 (0.166- 0.270)	3.416[3.177-3.666]	17.556[17.021- 18.101]	18.496[17.965-19.036]
**Gender and MSM**				
Female	0.013 [0.001-0.058]	0.217[0.116 -0.365]	4.378 [3.900 -4.894]	9.401 [8.716- 10.122]
Men were MSM	0.333[0.258 -0.421]	5.005 [4.649- 5.379]	26.646[25.797 -27.513]	24.918[24.100 -25.754]
Men were not MSM	0.0252[0.001 -0.111]	0.7105[0.400 -1.150]	2.2855 [1.733 -2.944]	11.694[10.589 -12.873]
**Country of born**				
Australia	0.196[0.1328- 0.276]	3.469[3.120 -3.842]	18.973[18.158- 19.811]	18.273[17.505-19.063]
Overseas	0.230[0.161 -0.316]	3.376[3.042 -3.733]	16.407[15.680- 17.155]	18.820[18.063-19.598]
**PrEP use**				
No	0.215[0.167 -0.272]	3.130[2.897 -3.375]	16.520[15.990 -17.061]	17.525[16.999- 18.063]
Yes	0.189[0.031 -0.583]	9.860 [7.980- 12.014]	40.706 [36.860 -44.811]	42.155 [38.210- 46.363]
**Age group**				
< 25 years old	0.1256[0.060- 0.226]	2.616[2.196 -3.086]	17.893[16.809 -19.024]	20.265[19.152-21.421]

25 - 34 years old	0.213[0.147 -0.295]	3.446[3.106- 3.811]	19.016[18.220- 19.835]	18.991[18.224 -19.779]
≥ 35 years old	0.290[0.190- 0.418]	4.017[3.539- 4.536]	14.672[13.748 -15.636]	15.980[15.037- 16.962]

Development of machine learning models using all predictors

Table S4. The area under ROC curve (AUC) of all predictors for predicting HIV over the next 12 months on testing data.

Models	AUC(Mean/95%CI)		
Bagged FDA	0.675	0.564	0.786
Bagged FDA using gCV Pruning	0.689	0.577	0.800
Bagged MARS using gCV Pruning	0.692	0.577	0.807
Boosted GLM	0.734	0.629	0.838
CIRF	0.731	0.615	0.847
KNN	0.686	0.559	0.813
MLR	0.677	0.543	0.810
SVM (Linear)	0.702	0.577	0.826
Kernel SVM (Polynomial)	0.727	0.616	0.838
Kernel SVM (RBF)	0.637	0.520	0.754

ENR	0.719	0.591	0.847
NB	0.718	0.598	0.837
RF	0.608	0.487	0.728
GBM	0.598	0.478	0.718
ensemble ENR+GBM+RF	0.608	0.486	0.730
XGBoost	0.718	0.618	0.817
MLP	0.614	0.484	0.745

Abb: Bagged FDA: Bagged Flexible Discriminant Analysis; Bagged FDA using gCV Pruning: Bagged Flexible Discriminant Analysis using Generalised Cross-Validation; Bagged MARS using gCV Pruning: Bagged Multivariate Adaptive Regression Splines using Generalized Cross-Validation; Boosted GLM: Boosted Generalized Linear Model; CIRF: Conditional Inference Random Forest; KNN: K-Nearest Neighbours; MLR: Multivariate Logistic Regression; SVM (Linear): Linear Support Vector Machines (without kernel extensions); Kernel SVM (Polynomial): SVM with a Polynomial Basis Kernel; Kernel SVM (RBF): SVM with a Radial Basis Function Kernel; ENR: Elastic-Net Regression; NB: Naïve Bayes; RF: Random Forest; GBM: Gradient Boosting Machines; XGBoost: eXtreme Gradient Boosting; MLP: multi-layer perceptron.

Table S5. The area under ROC curve (AUC) of all predictors for predicting syphilis over the next 12 months on testing data.

Models	AUC(Mean/95%CI)		
Bagged FDA	0.755	0.721	0.788

Bagged FDA using gCV Pruning	0.744	0.709	0.779
Bagged MARS using gCV Pruning	0.744	0.709	0.780
Boosted GLM	0.758	0.724	0.791
CIRF	0.745	0.711	0.779
KNN	0.631	0.591	0.671
MLR	0.747	0.713	0.782
SVM (Linear)	0.746	0.710	0.781
Kernel SVM (Polynomial)	0.740	0.705	0.776
Kernel SVM (RBF)	0.728	0.692	0.764
ENR	0.744	0.709	0.779
NB	0.730	0.694	0.765
RF	0.745	0.710	0.779
GBM	0.742	0.707	0.776
ensemble ENR+GBM+RF	0.749	0.715	0.783
XGBoost	0.741	0.714	0.767

MLP	0.567	0.526	0.608

Abb: Bagged FDA: Bagged Flexible Discriminant Analysis; Bagged FDA using gCV Pruning: Bagged Flexible Discriminant Analysis using Generalised Cross-Validation; Bagged MARS using gCV Pruning: Bagged Multivariate Adaptive Regression Splines using Generalized Cross-Validation; Boosted GLM: Boosted Generalized Linear Model; CIRF: Conditional Inference Random Forest; KNN: K-Nearest Neighbours; MLR: Multivariate Logistic Regression; SVM (Linear): Linear Support Vector Machines (without kernel extensions); Kernel SVM (Polynomial): SVM with a Polynomial Basis Kernel; Kernel SVM (RBF): SVM with a Radial Basis Function Kernel; ENR: Elastic-Net Regression; NB: Naïve Bayes; RF: Random Forest; GBM: Gradient Boosting Machines; XGBoost: eXtreme Gradient Boosting; MLP: multi-layer perceptron.

Table S6. The area under ROC curve (AUC) of all predictors for predicting gonorrhoea over the next 12 months on testing data.

Models	AUC(Mean/95%CI)		
Bagged FDA	0.714	0.698	0.730
Bagged FDA using gCV Pruning	0.720	0.704	0.737
Bagged MARS using gCV Pruning	0.721	0.705	0.737
Boosted GLM	0.719	0.703	0.735
CIRF	0.721	0.705	0.737
KNN	0.677	0.660	0.694
MLR	0.720	0.704	0.736
SVM (Linear)	0.698	0.681	0.714

Kernel SVM (Polynomial)	0.701	0.685	0.717
Kernel SVM (RBF)	0.703	0.687	0.719
ENR	0.721	0.705	0.737
NB	0.693	0.676	0.710
RF	0.721	0.705	0.737
GBM	0.720	0.704	0.736
ensemble ENR+GBM+RF	0.726	0.710	0.741
XGBoost	0.721	0.709	0.734
MLP	0.591	0.578	0.603

Abb: Bagged FDA: Bagged Flexible Discriminant Analysis; Bagged FDA using gCV Pruning: Bagged Flexible Discriminant Analysis using Generalised Cross-Validation; Bagged MARS using gCV Pruning: Bagged Multivariate Adaptive Regression Splines using Generalized Cross-Validation; Boosted GLM: Boosted Generalized Linear Model; CIRF: Conditional Inference Random Forest; KNN: K-Nearest Neighbours; MLR: Multivariate Logistic Regression; SVM (Linear): Linear Support Vector Machines (without kernel extensions); Kernel SVM (Polynomial): SVM with a Polynomial Basis Kernel; Kernel SVM (RBF): SVM with a Radial Basis Function Kernel; ENR: Elastic-Net Regression; NB: Naïve Bayes; RF: Random Forest; GBM: Gradient Boosting Machines; XGBoost: eXtreme Gradient Boosting; MLP: multi-layer perceptron.

Table S7. The area under ROC curve (AUC) of all predictors for predicting chlamydia over the next 12 months on testing data.

Models	AUC(Mean/95%CI)		
Bagged FDA	0.656	0.639	0.673

Bagged FDA using gCV Pruning	0.659	0.642	0.676
Bagged MARS using gCV Pruning	0.659	0.642	0.676
Boosted GLM	0.658	0.641	0.676
CIRF	0.659	0.642	0.677
KNN	0.620	0.602	0.638
MLR	0.658	0.641	0.675
SVM (Linear)	0.650	0.632	0.667
Kernel SVM (Polynomial)	0.649	0.631	0.667
Kernel SVM (RBF)	0.646	0.628	0.663
ENR	0.660	0.642	0.677
NB	0.640	0.622	0.658
RF	0.659	0.641	0.676
GBM	0.660	0.643	0.677
ensemble ENR+GBM+RF	0.663	0.646	0.680
XGBoost	0.662	0.648	0.675

MLP	0.513	0.501	0.524

Abb: Bagged FDA: Bagged Flexible Discriminant Analysis; Bagged FDA using gCV Pruning: Bagged Flexible Discriminant Analysis using Generalised Cross-Validation; Bagged MARS using gCV Pruning: Bagged Multivariate Adaptive Regression Splines using Generalized Cross-Validation; Boosted GLM: Boosted Generalized Linear Model; CIRF: Conditional Inference Random Forest; KNN: K-Nearest Neighbours; MLR: Multivariate Logistic Regression; SVM (Linear): Linear Support Vector Machines (without kernel extensions); Kernel SVM (Polynomial): SVM with a Polynomial Basis Kernel; Kernel SVM (RBF): SVM with a Radial Basis Function Kernel; ENR: Elastic-Net Regression; NB: Naïve Bayes; RF: Random Forest; GBM: Gradient Boosting Machines; XGBoost: eXtreme Gradient Boosting; MLP: multi-layer perceptron.

Table S8. Sensitivity of all predictors for predicting HIV over the next 12 months on testing data.

Models	Sensitivity (Mean/95%CI)		
Bagged FDA	0.614	0.606	0.623
Bagged FDA using gCV Pruning	0.747	0.739	0.754
Bagged MARS using gCV Pruning	0.033	0.030	0.036
Boosted GLM	0.561	0.553	0.570
CIRF	0.559	0.551	0.568
KNN	0.522	0.513	0.531
MLR	0.609	0.601	0.617
SVM (Linear)	0.634	0.625	0.642

Kernel SVM (Polynomial)	0.585	0.576	0.593
Kernel SVM (RBF)	0.603	0.595	0.611
ENR	0.571	0.562	0.579
NB	0.534	0.526	0.543
RF	0.621	0.613	0.629
GBM	0.596	0.588	0.604
ensemble ENR+GBM+RF	0.616	0.607	0.624
XGBoost	0.633	0.625	0.641
MLP	0.292	0.284	0.300

Abb: Bagged FDA: Bagged Flexible Discriminant Analysis; Bagged FDA using gCV Pruning: Bagged Flexible Discriminant Analysis using Generalised Cross-Validation; Bagged MARS using gCV Pruning: Bagged Multivariate Adaptive Regression Splines using Generalized Cross-Validation; Boosted GLM: Boosted Generalized Linear Model; CIRF: Conditional Inference Random Forest; KNN: K-Nearest Neighbours; MLR: Multivariate Logistic Regression; SVM (Linear): Linear Support Vector Machines (without kernel extensions); Kernel SVM (Polynomial): SVM with a Polynomial Basis Kernel; Kernel SVM (RBF): SVM with a Radial Basis Function Kernel; ENR: Elastic-Net Regression; NB: Naïve Bayes; RF: Random Forest; GBM: Gradient Boosting Machines; XGBoost: eXtreme Gradient Boosting; MLP: multi-layer perceptron.

Table S9. Sensitivity of all predictors for predicting syphilis over the next 12 months on testing data.

Models	Sensitivity (Mean/95%CI)		
Bagged FDA	0.570	0.560	0.579
Bagged FDA using gCV Pruning	0.582	0.573	0.591
Bagged MARS using gCV Pruning	0.044	0.040	0.048
Boosted GLM	0.561	0.551	0.570
CIRF	0.628	0.619	0.637
KNN	0.456	0.447	0.465
MLR	0.564	0.555	0.573
SVM (Linear)	0.569	0.560	0.578
Kernel SVM (Polynomial)	0.553	0.544	0.562
Kernel SVM (RBF)	0.581	0.572	0.590
ENR	0.549	0.540	0.558
NB	0.501	0.492	0.510
RF	0.628	0.619	0.637
GBM	0.621	0.612	0.630

ensemble ENR+GBM+RF	0.615	0.606	0.624
XGBoost	0.585	0.578	0.592
MLP	0.239	0.231	0.247

Abb: Bagged FDA: Bagged Flexible Discriminant Analysis; Bagged FDA using gCV Pruning: Bagged Flexible Discriminant Analysis using Generalised Cross-Validation; Bagged MARS using gCV Pruning: Bagged Multivariate Adaptive Regression Splines using Generalized Cross-Validation; Boosted GLM: Boosted Generalized Linear Model; CIRF: Conditional Inference Random Forest; KNN: K-Nearest Neighbours; MLR: Multivariate Logistic Regression; SVM (Linear): Linear Support Vector Machines (without kernel extensions); Kernel SVM (Polynomial): SVM with a Polynomial Basis Kernel; Kernel SVM (RBF): SVM with a Radial Basis Function Kernel; ENR: Elastic-Net Regression; NB: Naïve Bayes; RF: Random Forest; GBM: Gradient Boosting Machines; XGBoost: eXtreme Gradient Boosting; MLP: multi-layer perceptron.

Table S10. Sensitivity of all predictors for predicting gonorrhoea over the next 12 months on testing data.

Models	Sensitivity (Mean/95%CI)		
Bagged FDA	0.546	0.536	0.555
Bagged FDA using gCV Pruning	0.548	0.539	0.557
Bagged MARS using gCV Pruning	0.073	0.068	0.077
Boosted GLM	0.547	0.537	0.556
CIRF	0.615	0.606	0.624
KNN	0.515	0.505	0.524

MLR	0.543	0.534	0.553
SVM (Linear)	0.524	0.515	0.533
Kernel SVM (Polynomial)	0.538	0.528	0.547
Kernel SVM (RBF)	0.569	0.559	0.578
ENR	0.545	0.536	0.554
NB	0.494	0.485	0.503
RF	0.614	0.605	0.623
GBM	0.606	0.597	0.615
ensemble ENR+GBM+RF	0.600	0.590	0.609
XGBoost	0.567	0.560	0.574
MLP	0.538	0.529	0.547

Abb: Bagged FDA: Bagged Flexible Discriminant Analysis; Bagged FDA using gCV Pruning: Bagged Flexible Discriminant Analysis using Generalised Cross-Validation; Bagged MARS using gCV Pruning: Bagged Multivariate Adaptive Regression Splines using Generalized Cross-Validation; Boosted GLM: Boosted Generalized Linear Model; CIRF: Conditional Inference Random Forest; KNN: K-Nearest Neighbours; MLR: Multivariate Logistic Regression; SVM (Linear): Linear Support Vector Machines (without kernel extensions); Kernel SVM (Polynomial): SVM with a Polynomial Basis Kernel; Kernel SVM (RBF): SVM with a Radial Basis Function Kernel; ENR: Elastic-Net Regression; NB: Naïve Bayes; RF: Random Forest; GBM: Gradient Boosting Machines; XGBoost: eXtreme Gradient Boosting; MLP: multi-layer perceptron.

Table S11. Sensitivity of all predictors for predicting chlamydia over the next 12 months on testing data.

Models	Sensitivity (Mean/95%CI)		
Bagged FDA	0.420	0.411	0.429
Bagged FDA using gCV Pruning	0.425	0.416	0.433
Bagged MARS using gCV Pruning	0.088	0.083	0.093
Boosted GLM	0.416	0.407	0.425
CIRF	0.510	0.501	0.519
KNN	0.401	0.392	0.410
MLR	0.418	0.410	0.427
SVM (Linear)	0.400	0.391	0.409
Kernel SVM (Polynomial)	0.402	0.393	0.411
Kernel SVM (RBF)	0.416	0.408	0.425
ENR	0.418	0.409	0.426
NB	0.378	0.370	0.387
RF	0.523	0.514	0.532

GBM	0.465	0.456	0.474
ensemble ENR+GBM+RF	0.505	0.496	0.514
XGBoost	0.483	0.476	0.490
MLP	0.600	0.592	0.609

Abb: Bagged FDA: Bagged Flexible Discriminant Analysis; Bagged FDA using gCV Pruning: Bagged Flexible Discriminant Analysis using Generalised Cross-Validation; Bagged MARS using gCV Pruning: Bagged Multivariate Adaptive Regression Splines using Generalized Cross-Validation; Boosted GLM: Boosted Generalized Linear Model; CIRF: Conditional Inference Random Forest; KNN: K-Nearest Neighbours; MLR: Multivariate Logistic Regression; SVM (Linear): Linear Support Vector Machines (without kernel extensions); Kernel SVM (Polynomial): SVM with a Polynomial Basis Kernel; Kernel SVM (RBF): SVM with a Radial Basis Function Kernel; ENR: Elastic-Net Regression; NB: Naïve Bayes; RF: Random Forest; GBM: Gradient Boosting Machines; XGBoost: eXtreme Gradient Boosting; MLP: multi-layer perceptron.

Table S12. Specificity of all predictors for predicting HIV over the next 12 months on testing data.

Models	Specificity (Mean/95%CI)		
Bagged FDA	0.564	0.346	0.782
Bagged FDA using gCV Pruning	0.359	0.113	0.605
Bagged MARS using gCV Pruning	0.923	0.778	1.068
Boosted GLM	0.872	0.699	1.045

CIRF	0.692	0.441	0.943
KNN	0.750	0.505	0.995
MLR	0.641	0.381	0.901
SVM (Linear)	0.641	0.381	0.901
Kernel SVM (Polynomial)	0.718	0.497	0.939
Kernel SVM (RBF)	0.564	0.297	0.831
ENR	0.769	0.540	0.998
NB	0.744	0.507	0.980
RF	0.487	0.216	0.758
GBM	0.462	0.191	0.733
ensemble ENR+GBM+RF	0.462	0.193	0.730
XGBoost	0.686	0.432	0.939
MLP	0.531	0.241	0.821

Abb: Bagged FDA: Bagged Flexible Discriminant Analysis; Bagged FDA using gCV Pruning: Bagged Flexible Discriminant Analysis using Generalised Cross-Validation; Bagged MARS using gCV Pruning: Bagged Multivariate Adaptive Regression Splines using Generalized Cross-Validation; Boosted GLM: Boosted Generalized Linear Model; CIRF: Conditional Inference Random Forest; KNN: K-Nearest Neighbours; MLR: Multivariate Logistic Regression; SVM (Linear): Linear Support Vector Machines (without kernel extensions); Kernel SVM (Polynomial): SVM

with a Polynomial Basis Kernel; Kernel SVM (RBF): SVM with a Radial Basis Function Kernel; ENR: Elastic-Net Regression; NB: Naïve Bayes; RF: Random Forest; GBM: Gradient Boosting Machines; XGBoost: eXtreme Gradient Boosting; MLP: multi-layer perceptron.

Table S13. Specificity of all predictors for predicting syphilis over the next 12 months on testing data.

Models	Specificity (Mean/95%CI)		
Bagged FDA	0.779	0.712	0.846
Bagged FDA using gCV Pruning	0.754	0.685	0.822
Bagged MARS using gCV Pruning	0.810	0.748	0.873
Boosted GLM	0.800	0.736	0.863
CIRF	0.705	0.633	0.778
KNN	0.715	0.643	0.787
MLR	0.779	0.713	0.845
SVM (Linear)	0.764	0.697	0.832
Kernel SVM (Polynomial)	0.780	0.715	0.846
Kernel SVM (RBF)	0.732	0.661	0.802
ENR	0.784	0.718	0.849
NB	0.798	0.735	0.862

RF	0.705	0.633	0.778
GBM	0.709	0.637	0.781
ensemble ENR+GBM+RF	0.735	0.665	0.805
XGBoost	0.735	0.680	0.789
MLP	0.885	0.835	0.936

Abb: Bagged FDA: Bagged Flexible Discriminant Analysis; Bagged FDA using gCV Pruning: Bagged Flexible Discriminant Analysis using Generalised Cross-Validation; Bagged MARS using gCV Pruning: Bagged Multivariate Adaptive Regression Splines using Generalized Cross-Validation; Boosted GLM: Boosted Generalized Linear Model; CIRF: Conditional Inference Random Forest; KNN: K-Nearest Neighbours; MLR: Multivariate Logistic Regression; SVM (Linear): Linear Support Vector Machines (without kernel extensions); Kernel SVM (Polynomial): SVM with a Polynomial Basis Kernel; Kernel SVM (RBF): SVM with a Radial Basis Function Kernel; ENR: Elastic-Net Regression; NB: Naïve Bayes; RF: Random Forest; GBM: Gradient Boosting Machines; XGBoost: eXtreme Gradient Boosting; MLP: multi-layer perceptron.

Table S14. Specificity of all predictors for predicting gonorrhoea over the next 12 months on testing data.

Models	Specificity (Mean/95%CI)		
Bagged FDA	0.787	0.759	0.815
Bagged FDA using gCV Pruning	0.791	0.763	0.819
Bagged MARS using gCV Pruning	0.804	0.777	0.831
Boosted GLM	0.793	0.765	0.821

CIRF	0.715	0.684	0.746
KNN	0.763	0.734	0.792
MLR	0.787	0.759	0.816
SVM (Linear)	0.791	0.763	0.819
Kernel SVM (Polynomial)	0.781	0.753	0.810
Kernel SVM (RBF)	0.741	0.711	0.771
ENR	0.798	0.770	0.825
NB	0.801	0.773	0.828
RF	0.707	0.676	0.738
GBM	0.712	0.681	0.743
ensemble ENR+GBM+RF	0.731	0.700	0.761
XGBoost	0.772	0.749	0.794
MLP	0.645	0.620	0.671

Abb: Bagged FDA: Bagged Flexible Discriminant Analysis; Bagged FDA using gCV Pruning: Bagged Flexible Discriminant Analysis using Generalised Cross-Validation; Bagged MARS using gCV Pruning: Bagged Multivariate Adaptive Regression Splines using Generalized Cross-Validation; Boosted GLM: Boosted Generalized Linear Model; CIRF: Conditional Inference Random Forest; KNN: K-Nearest Neighbours; MLR: Multivariate Logistic Regression; SVM (Linear): Linear Support Vector Machines (without kernel extensions); Kernel SVM (Polynomial): SVM

with a Polynomial Basis Kernel; Kernel SVM (RBF): SVM with a Radial Basis Function Kernel; ENR: Elastic-Net Regression; NB: Naïve Bayes; RF: Random Forest; GBM: Gradient Boosting Machines; XGBoost: eXtreme Gradient Boosting; MLP: multi-layer perceptron.

Table S15. Specificity of all predictors for predicting chlamydia over the next 12 months on testing data.

Models	Specificity (Mean/95%CI)		
Bagged FDA	0.794	0.768	0.820
Bagged FDA using gCV Pruning	0.791	0.765	0.818
Bagged MARS using gCV Pruning	0.809	0.784	0.834
Boosted GLM	0.795	0.769	0.822
CIRF	0.716	0.687	0.745
KNN	0.769	0.742	0.796
MLR	0.791	0.764	0.817
SVM (Linear)	0.797	0.771	0.823
Kernel SVM (Polynomial)	0.795	0.769	0.821
Kernel SVM (RBF)	0.781	0.754	0.808
ENR	0.797	0.771	0.823
NB	0.798	0.772	0.824

RF	0.699	0.669	0.728
GBM	0.763	0.735	0.790
ensemble ENR+GBM+RF	0.724	0.695	0.753
XGBoost	0.739	0.717	0.761
MLP	0.439	0.401	0.477

Abb: Bagged FDA: Bagged Flexible Discriminant Analysis; Bagged FDA using gCV Pruning: Bagged Flexible Discriminant Analysis using Generalised Cross-Validation; Bagged MARS using gCV Pruning: Bagged Multivariate Adaptive Regression Splines using Generalized Cross-Validation; Boosted GLM: Boosted Generalized Linear Model; CIRF: Conditional Inference Random Forest; KNN: K-Nearest Neighbours; MLR: Multivariate Logistic Regression; SVM (Linear): Linear Support Vector Machines (without kernel extensions); Kernel SVM (Polynomial): SVM with a Polynomial Basis Kernel; Kernel SVM (RBF): SVM with a Radial Basis Function Kernel; ENR: Elastic-Net Regression; NB: Naïve Bayes; RF: Random Forest; GBM: Gradient Boosting Machines; XGBoost: eXtreme Gradient Boosting; MLP: multi-layer perceptron.

Table S16. F1 of all predictors for predicting HIV over the next 12 months on testing data.

Models	F1 (Mean/95%CI)		
Bagged FDA	0.614	0.606	0.623
Bagged FDA using gCV Pruning	0.746	0.739	0.754
Bagged MARS using gCV Pruning	0.034	0.031	0.037

Boosted GLM	0.562	0.553	0.570
CIRF	0.559	0.551	0.568
KNN	0.522	0.513	0.531
MLR	0.609	0.601	0.617
SVM (Linear)	0.634	0.625	0.642
Kernel SVM (Polynomial)	0.585	0.576	0.593
Kernel SVM (RBF)	0.603	0.595	0.611
ENR	0.571	0.563	0.580
NB	0.534	0.526	0.543
RF	0.621	0.612	0.629
GBM	0.596	0.587	0.604
ensemble ENR+GBM+RF	0.616	0.607	0.624
XGBoost	0.633	0.625	0.641
MLP	0.293	0.285	0.300

Abb: Bagged FDA: Bagged Flexible Discriminant Analysis; Bagged FDA using gCV Pruning: Bagged Flexible Discriminant Analysis using Generalised Cross-Validation; Bagged MARS using gCV Pruning: Bagged Multivariate Adaptive Regression Splines using Generalized Cross-Validation; Boosted GLM: Boosted Generalized Linear Model; CIRF: Conditional Inference Random Forest; KNN: K-Nearest Neighbours; MLR:

Multivariate Logistic Regression; SVM (Linear): Linear Support Vector Machines (without kernel extensions); Kernel SVM (Polynomial): SVM with a Polynomial Basis Kernel; Kernel SVM (RBF): SVM with a Radial Basis Function Kernel; ENR: Elastic-Net Regression; NB: Naïve Bayes; RF: Random Forest; GBM: Gradient Boosting Machines; XGBoost: eXtreme Gradient Boosting; MLP: multi-layer perceptron.

Table S17. F1 of all predictors for predicting syphilis over the next 12 months on testing data.

Models	F1 (Mean/95%CI)		
Bagged FDA	0.572	0.563	0.581
Bagged FDA using gCV Pruning	0.585	0.575	0.594
Bagged MARS using gCV Pruning	0.054	0.050	0.058
Boosted GLM	0.564	0.555	0.573
CIRF	0.629	0.620	0.638
KNN	0.459	0.450	0.468
MLR	0.567	0.558	0.576
SVM (Linear)	0.572	0.563	0.581
Kernel SVM (Polynomial)	0.556	0.547	0.565
Kernel SVM (RBF)	0.583	0.574	0.592
ENR	0.552	0.543	0.561

NB	0.505	0.496	0.514
RF	0.629	0.620	0.638
GBM	0.622	0.614	0.631
ensemble ENR+GBM+RF	0.616	0.607	0.625
XGBoost	0.587	0.580	0.594
MLP	0.248	0.239	0.256

Abb: Bagged FDA: Bagged Flexible Discriminant Analysis; Bagged FDA using gCV Pruning: Bagged Flexible Discriminant Analysis using Generalised Cross-Validation; Bagged MARS using gCV Pruning: Bagged Multivariate Adaptive Regression Splines using Generalized Cross-Validation; Boosted GLM: Boosted Generalized Linear Model; CIRF: Conditional Inference Random Forest; KNN: K-Nearest Neighbours; MLR: Multivariate Logistic Regression; SVM (Linear): Linear Support Vector Machines (without kernel extensions); Kernel SVM (Polynomial): SVM with a Polynomial Basis Kernel; Kernel SVM (RBF): SVM with a Radial Basis Function Kernel; ENR: Elastic-Net Regression; NB: Naïve Bayes; RF: Random Forest; GBM: Gradient Boosting Machines; XGBoost: eXtreme Gradient Boosting; MLP: multi-layer perceptron.

Table S18. F1 of all predictors for predicting gonorrhoea over the next 12 months on testing data.

Models	F1 (Mean/95%CI)		
Bagged FDA	0.562	0.553	0.571
Bagged FDA using gCV Pruning	0.564	0.555	0.573

Bagged MARS using gCV Pruning	0.122	0.116	0.128
Boosted GLM	0.563	0.554	0.572
CIRF	0.622	0.613	0.631
KNN	0.531	0.522	0.540
MLR	0.560	0.551	0.569
SVM (Linear)	0.542	0.533	0.551
Kernel SVM (Polynomial)	0.554	0.545	0.563
Kernel SVM (RBF)	0.580	0.571	0.589
ENR	0.562	0.553	0.571
NB	0.514	0.506	0.523
RF	0.620	0.612	0.629
GBM	0.613	0.605	0.622
ensemble ENR+GBM+RF	0.608	0.600	0.617
XGBoost	0.581	0.574	0.587
MLP	0.555	0.543	0.568

Abb: Bagged FDA: Bagged Flexible Discriminant Analysis; Bagged FDA using gCV Pruning: Bagged Flexible Discriminant Analysis using Generalised Cross-Validation; Bagged MARS using gCV Pruning: Bagged Multivariate Adaptive Regression Splines using Generalized Cross-Validation; Boosted GLM: Boosted Generalized Linear Model; CIRF: Conditional Inference Random Forest; KNN: K-Nearest Neighbours; MLR: Multivariate Logistic Regression; SVM (Linear): Linear Support Vector Machines (without kernel extensions); Kernel SVM (Polynomial): SVM with a Polynomial Basis Kernel; Kernel SVM (RBF): SVM with a Radial Basis Function Kernel; ENR: Elastic-Net Regression; NB: Naïve Bayes; RF: Random Forest; GBM: Gradient Boosting Machines; XGBoost: eXtreme Gradient Boosting; MLP: multi-layer perceptron.

Table S19. F1 of all predictors for predicting chlamydia over the next 12 months on testing data.

Models	F1 (Mean/95%CI)		
Bagged FDA	0.447	0.438	0.456
Bagged FDA using gCV Pruning	0.451	0.442	0.460
Bagged MARS using gCV Pruning	0.140	0.134	0.146
Boosted GLM	0.443	0.435	0.452
CIRF	0.525	0.516	0.534
KNN	0.428	0.419	0.436
MLR	0.445	0.437	0.454
SVM (Linear)	0.429	0.420	0.437

Kernel SVM (Polynomial)	0.430	0.422	0.439
Kernel SVM (RBF)	0.443	0.434	0.451
ENR	0.445	0.436	0.453
NB	0.409	0.400	0.417
RF	0.535	0.527	0.544
GBM	0.486	0.478	0.495
ensemble ENR+GBM+RF	0.521	0.512	0.530
XGBoost	0.502	0.495	0.508
MLP	0.598	0.585	0.611

Abb: Bagged FDA: Bagged Flexible Discriminant Analysis; Bagged FDA using gCV Pruning: Bagged Flexible Discriminant Analysis using Generalised Cross-Validation; Bagged MARS using gCV Pruning: Bagged Multivariate Adaptive Regression Splines using Generalized Cross-Validation; Boosted GLM: Boosted Generalized Linear Model; CIRF: Conditional Inference Random Forest; KNN: K-Nearest Neighbours; MLR: Multivariate Logistic Regression; SVM (Linear): Linear Support Vector Machines (without kernel extensions); Kernel SVM (Polynomial): SVM with a Polynomial Basis Kernel; Kernel SVM (RBF): SVM with a Radial Basis Function Kernel; ENR: Elastic-Net Regression; NB: Naïve Bayes; RF: Random Forest; GBM: Gradient Boosting Machines; XGBoost: eXtreme Gradient Boosting; MLP: multi-layer perceptron.

Performance metrics of the 12-month HIV/STI risk prediction tool

Table S20. Performance metrics of the 12-month HIV/STI risk prediction tool (Best machine learning models using selected predictors).

	Mean	95%CI	
**HIV**			
AUC	0.722	0.609	0.834
Sensitivity	0.597	0.589	0.606
Specificity	0.738	0.516	0.961
F1	0.597	0.589	0.606
**syphilis**			
AUC	0.754	0.719	0.788
Sensitivity	0.611	0.602	0.621
Specificity	0.780	0.710	0.851
F1	0.613	0.604	0.623
**gonorrhoea**			
AUC	0.730	0.714	0.746
Sensitivity	0.590	0.580	0.599
Specificity	0.764	0.734	0.793

F1	0.601	0.592	0.610
**chlamydia**			
AUC	0.666	0.649	0.683
Sensitivity	0.494	0.485	0.503
Specificity	0.741	0.713	0.770
F1	0.511	0.503	0.520

Table S21. The performance comparison of the best machine learning models using all predictors and risk prediction tool.

	AUC(Mean/95%CI)
**HIV**	
Best ML using all predictors	0.734[0.629-0.838]
Risk prediction tool (Best ML using selected predictors)	0.722[0.609-0.834]
**syphilis**	
Best ML using all predictors	0.758[0.724-0.791]
Risk prediction tool (Best ML using selected predictors)	0.754[0.719-0.788]
**gonorrhea**	
Best ML using all predictors	0.726[0.710-0.741]

Risk prediction tool (Best ML using selected predictors)	0.730[0.714-0.746]
**chlamydia**	
Best ML using all predictors	0.663[0.646-0.680]
Risk prediction tool (Best ML using selected predictors)	0.666[0.649-0.683]

ML= machine learning.

12-month HIV/STI risk estimate

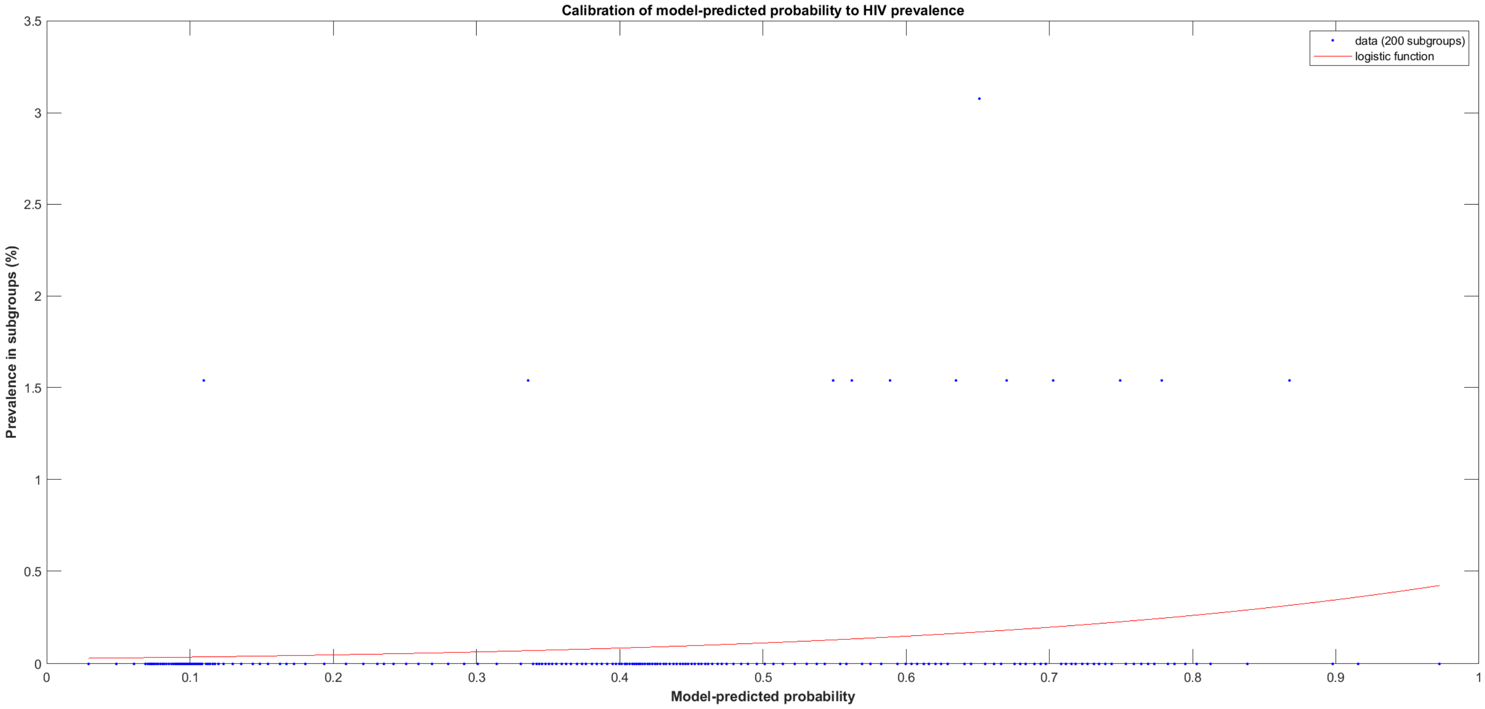

Figure S2. The fitting curve of model-predicted probability and HIV prevalence over the next 12 months. The logistic function assumes the form $f(x) = L/(1 + e^{-r(x-x_0)})$, where L =50.05, r =2.81 and x_0 = 2.668.

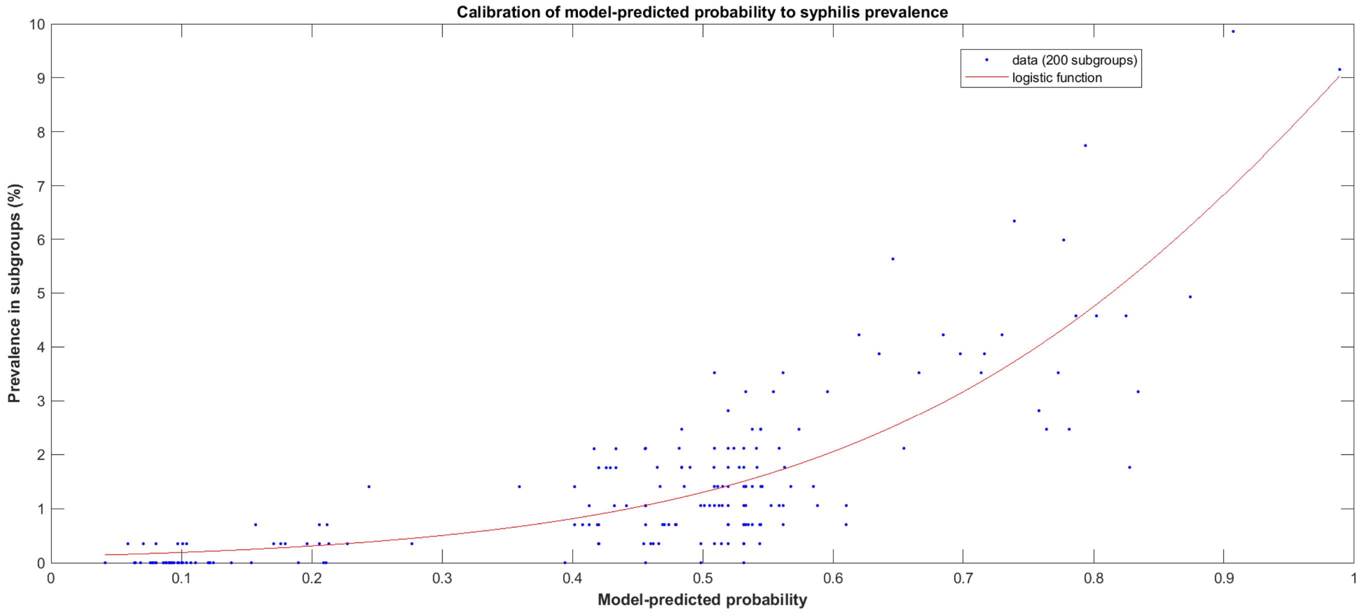

Figure S3. The fitting curve of model-predicted probability and syphilis prevalence over the next 12 months. The logistic function assumes the form $\boldsymbol{f(x) = L/(1 + e^{-r(x-x_0)})}$, where $L = 21.89$, $r = 4.931$ and $x_0 = 1.06$.

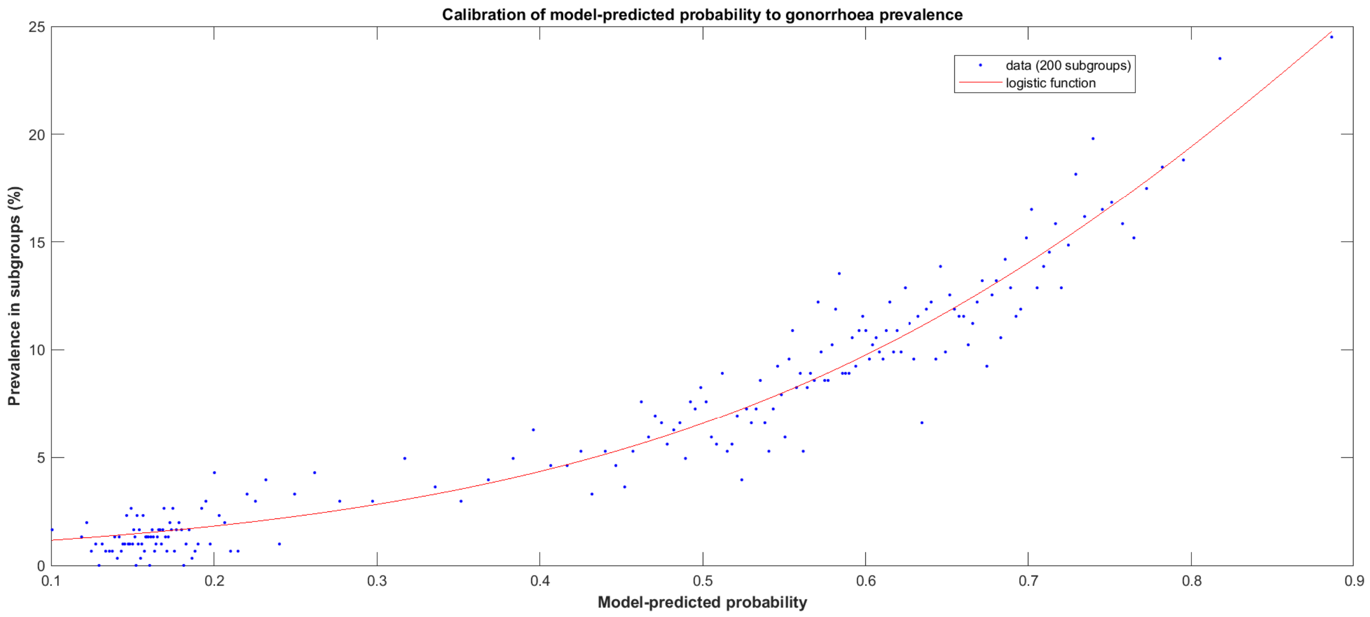

Figure S4. The fitting curve of model-predicted probability and gonorrhoea prevalence over the next 12 months. The logistic function assumes the form $\boldsymbol{f(x) = L/(1 + e^{-r(x-x_0)})}$, where L =57.15, r =4.587 and x_0 = 0.9446.

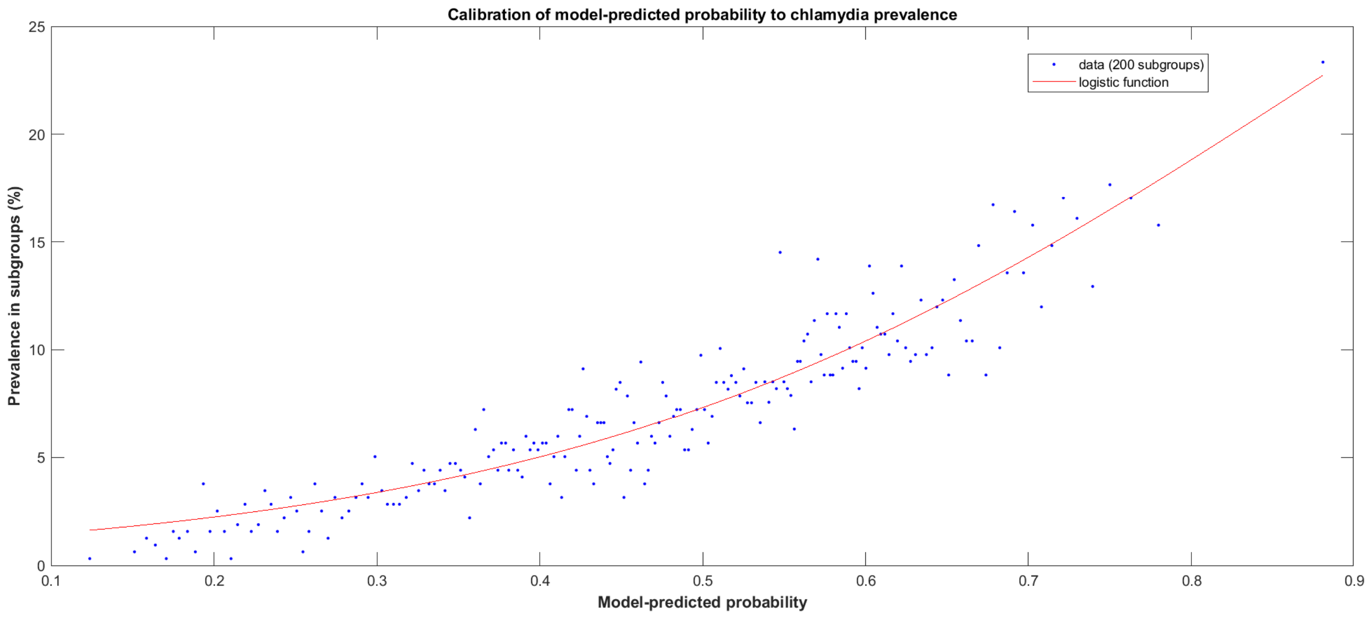

Figure S5. The fitting curve of model-predicted probability and chlamydia prevalence over the next 12 months. The logistic function assumes the form $\boldsymbol{f(x) = L/(1+e^{-r(x-x_0)})}$, where $L = 44.59$, $r = 4.377$ and $x_0 = 0.8718$.

User interface design

We used survey data to calculate a user' risk of HIV/STI over the next 12 months. The calibrated probability of HIV/STI over the next 12 months was divided into three risk levels: HIV (low [< 0.1%], medium [0.1-1.0%], and high [≥ 1.0 %]], syphilis (low [< 0.2%], medium [0.2-5.0%], and high [≥ 5.0%]), gonorrhoea (low [< 0.1%], medium [0.1-1.0%], and high [≥ 1.0%]), and chlamydia (low [< 2.0%], medium [2.0-15.0%], and high [≥ 15.0%]).

In the output section, our risk prediction tool could calculate a qualitative outline of risk over the next 12 months, e.g.,

1. *"Your HIV risk is about 2/1000 (moderate risk). Over next 12 months, in a group of 1000 people like me, 2 will have HIV. 998 people will not have HIV."*
2. *"Your syphilis risk is about 5/1000 (moderate risk). Over next 12 months, in a group of 1000 people like me, 5 will have syphilis. 995 people will not have syphilis."*
3. *"Your gonorrhoea risk is about 10/1000 (moderate risk). Over next 12 months, in a group of 1000 people like me, 10 will have gonorrhoea. 990 people will not have gonorrhoea."*
4. *"Your chlamydia risk is about 30/1000 (moderate risk). Over next 12 months, in a group of 1000 people like me, 30 will have chlamydia. 970 people will not have chlamydia."*

Reference

1. Selvey, L.A.; Slimings, C.; Adams, E.; Manuel, J. Incidence and predictors of HIV, chlamydia and gonorrhoea among men who have sex with men attending a peer-based clinic. *Sex Health* **2018**, *15*, 451-459, doi:10.1071/sh17181.
2. Vandormael, A.; Dobra, A.; Bärnighausen, T.; de Oliveira, T.; Tanser, F. Incidence rate estimation, periodic testing and the limitations of the mid-point imputation approach. *Int J Epidemiol* **2018**, *47*, 236-245, doi:10.1093/ije/dyx134.
3. Shehzad, A.; Rockwood, K.; Stanley, J.; Dunn, T.; Howlett, S.E. Use of Patient-Reported Symptoms from an Online Symptom Tracking Tool for Dementia Severity Staging: Development and Validation of a Machine Learning Approach. *Journal of medical Internet research* **2020**, *22*, e20840-e20840, doi:10.2196/20840.
4. Menardi, G.; Torelli, N. Training and assessing classification rules with imbalanced data. *Data Mining & Knowledge Discovery* **2014**, *28*, 92-122.
5. Liao, X.; Kerr, D.; Morales, J.; Duncan, I. Application of Machine Learning to Identify Clustering of Cardiometabolic Risk Factors in U.S. Adults. *Diabetes technology & therapeutics* **2019**, *21*, 245-253, doi:10.1089/dia.2018.0390.
